# BOPAM’s Bright and Dark Excited States: Insight from Structural, Photophysical, and Quantum Chemical Investigations

**DOI:** 10.3390/molecules30132673

**Published:** 2025-06-20

**Authors:** Kexin Yu, Thanh Chung Pham, Jianjun Huang, Yixuan Li, Luc Van Meervelt, Mark Van der Auweraer, Daniel Escudero, Wim Dehaen

**Affiliations:** 1 Sustainable Chemistry for Metals and Molecules, Department of Chemistry, KU Leuven, Celestijnenlaan 200F, 3001 Leuven, Belgium; kexin.yu@kuleuven.be (K.Y.); jianjun.huang@kuleuven.be (J.H.); 2 Quantum Chemistry and Physical Chemistry, Department of Chemistry, KU Leuven, Celestijnenlaan 200F, 3001 Leuven, Belgium; yixuan.li@kuleuven.be; 3 Biochemistry, Molecular and Structural Biology, Department of Chemistry, KU Leuven, Celestijnenlaan 200F, 3001 Leuven, Belgium; luc.vanmeervelt@kuleuven.be; 4 Molecular Imaging and Photonics, Department of Chemistry, KU Leuven, Celestijnenlaan 200F, 3001 Leuven, Belgium; mark.vanderauweraer@kuleuven.be

**Keywords:** BOPAM, dark state, charge transfer, donor–acceptor, fluorophore

## Abstract

BOPAM exhibits high fluorescence quantum yields, along with exceptional photostability, rendering it a promising platform for applications as fluorescence sensors. However, the development of BOPAM-based fluorophores with extended emission wavelengths remains limited, and the underlying mechanisms of fluorescence quenching via the population of dark twisted intramolecular charge transfer (^1^TICT) excited states are not yet fully understood. To address these gaps, we synthesized a series of BOPAM derivatives by incorporating electron-donating groups at the boron atoms and the phenyl rings of the BOPAM core. The introduction of bromide, phenyl, and naphthyl groups preserved the intrinsic locally excited (^1^LE) emission of BOPAM. In contrast, the incorporation of diphenylamine (**BP-DA**) and triphenylamine (**BP-TA**) moieties resulted in a red-shifted emission, attributed to an enhanced intramolecular charge transfer (ICT) process. Notably, in acetonitrile, **BP-DA** exhibited weak fluorescence originating from a ^1^TICT state, which was populated via the S_2_ → ^1^TICT transition. Furthermore, the emission observed from **BP-TA** was associated with a higher-lying excited state, likely the initially populated S_2_ state possessing a ^1^LE character. These findings not only introduce novel red-emissive BOPAM-based fluorophores, but also offer valuable insights into the role of the S_2_ state in governing fluorescence quenching mechanisms in BOPAM derivatives.

## 1. Introduction

In recent years, fluorophores have shown broad application in fields such as bioimaging, sensors, and organic optoelectronics, significantly driving the exploration and development of novel organic fluorescent dyes [1,2,3,4,5,6]. Particularly, organic small-molecule fluorescent dyes, with their high synthetic flexibility, exhibit remarkable advantages in molecular structure design and functional modifications. Since boron dipyrromethene (BODIPY) fluorescent dyes were first reported by Treibs and Kreuzer in 1968, research on boron-chelated small-molecule dyes has continued to flourish [7,8,9,10]. The success of BODIPYs can be attributed not only to their excellent photophysical properties, but also to their rich potential for functional modifications [11,12]. The core structure of BODIPY has multiple modification sites, allowing for functionalization at each position of the central skeleton through straightforward chemical reactions such as coupling reactions, Knoevenagel condensation, and halogenation [13,14]. These modifications enable the improvement in various physicochemical properties including water solubility and biocompatibility [13,14,15,16,17,18,19].

A recently reported novel asymmetric diboron complex small-molecule dye, bis(difluoroboron)pyrrole amidrazone (BOPAM), features a central core composed of a pyrrole unit, a six-membered and a five-membered heterocyclic ring, maintaining excellent overall planarity [20]. BOPAM demonstrates highly desirable photophysical properties, including a high fluorescence quantum yield (FLQY) in both organic solvents and the solid state, along with a straightforward synthesis, structural tunability, and significant stability [20,21]. These promising characteristics enhance the potential of BOPAM for practical applications such as acid sensing [21]. However, for broader applicability, particularly in optoelectronic and bioimaging purposes, its emission should be close to the red or near-infrared (NIR) region while achieving a large Stokes shift. This spectral tuning can be realized through molecular modifications, specifically by introducing strong electron-donating groups (EDGs). Thanks to the BOPAM core’s intrinsic strength as an electron-withdrawing group (EWG), establishing a donor–acceptor framework is straightforward in achieving the desired, red-shifted emission and increased Stokes shift.

**Figure 1:**
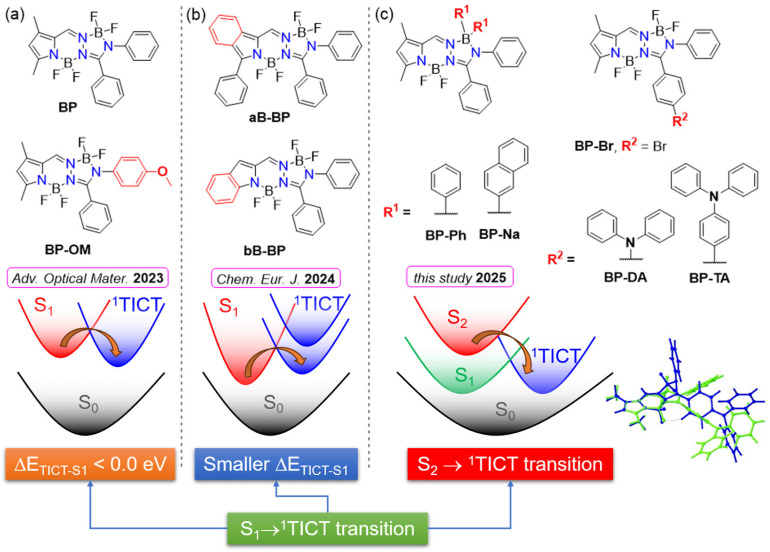
Structures of **BOPAM** and excited state decay models highlighting the role of the ^1^TICT state in fluorescence quenching: (**a**) **BP** (**5a**) and **BP-OM** (**5i**) [20]; (**b**) **aB-BP** (**5a**) and **bB-BP** (**5**) [21]; (**c**) in the current study: **BP-R^1^** and **BP-R^2^**. Overlay of the optimized geometries of ^1^ICT (green) and ^1^TICT (blue) states of **BP-DA** are shown in the right-bottom of the figure.

Therefore, we systematically introduced phenyl (Ph) and naphthyl (Na) groups onto the boron center, yielding **BP-Ph** and **BP-Na**, respectively. Subsequently, bromine (Br), diphenylamine (DA), and triphenylamine (TA) groups were incorporated into the phenyl ring of the BOPAM core to generate **BP-Br**, **BP-DA**, and **BP-TA**, respectively (see Figure 1). Single-crystal X-ray diffraction (SC-XRD) analysis was conducted, revealing distinct emission characteristics in the solid state including the intrinsic localized excited (^1^LE) emission of BOPAM as well as twisted configurations and charge transfer-induced emissions. In organic solvents, **BP-Ph**, **BP-Na**, and **BP-Br** predominantly exhibit ^1^LE emissions with high FLQY comparable to the parent BOPAM (**BP**). In contrast, **BP-DA** and **BP-TA** display characteristic intramolecular charge transfer (ICT) emissions in toluene and tetrahydrofuran, resulting in a red-shifted emission relative to **BP**. Notably, **BP-TA** exhibits a broad-band emission centered at 608 nm with a high FLQY of 51% in tetrahydrofuran. These findings provide a new molecular design of BOPAM-based fluorophores bearing red-shifted emissions combined with large Stokes shifts, demonstrating their potential for practical bioimaging applications.

Previous studies [20,21] have highlighted the role of a twisted intramolecular charge transfer (^1^TICT) state, corresponding to a local minimum in the S_1_ potential energy surface, as a non-emissive state and have identified its pivotal role in the fluorescence quenching mechanisms of **BP-OM** [20] (Figure 1a) and **bB-BP** [21] (Figure 1b). Importantly, the optimized geometry of the ^1^TICT state adopts a twisted configuration, in contrast to the planar configurations typically associated with other excited states such as the ^1^ICT or ^1^LE states (see the ^1^TICT configuration of **BP-DA** in Figure 1c). Moreover, the ^1^TICT state is characterized by its small oscillator strength (f < 0.02) and red-shifted energy. The S_1_ → ^1^TICT transition is readily accessible due to either a negative energy gap [20] (Figure 1a) or a smaller energy gap [21] (Figure 1b) between the S_1_ and ^1^TICT states, both of which critically influence the observed quenching mechanisms. For the systems shown in Figure 1a,b, S_1_ is of ^1^LE character.

Within the series of the new investigated compounds (see Figure 1c), some of them still adhere to this model (e.g., **BP-Br**, **BP-Ph**, and **BP-Na**). However, in the case of **BP-DA**, bearing an ^1^ICT state as S_1_, the calculated S_1_-^1^TICT energy gap (0.49 eV) is substantially larger than those calculated for **BP**, **BP-Br, BP-Ph**, **BP-Na**, and **bB-BP** (0.10–0.28 eV). Despite the large energy gap, emission from the ^1^TICT state is still observed for **BP-DA**. Furthermore, the emission observed from **BP-TA** is associated with a higher-lying excited state, likely the initially populated S_2_ state. These findings suggest that a more complex photophysical scenario is obtained for **BP-DA** and **BP-TA**. Alternative and complementary models were devised here for BOPAM’s excited state decay based on our joint experimental/computational investigations.

## 2. Results and Discussion

### 2.1. Synthesis

**Scheme 1:**
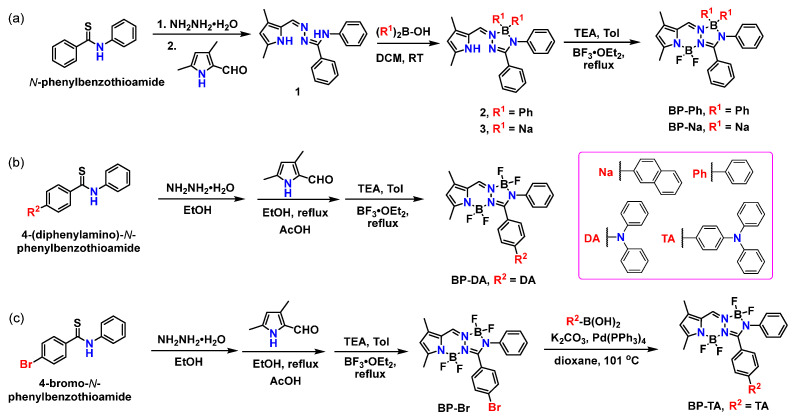
Synthetic route of (**a**) **BP-Ph** and **BP-Na**, (**b**) **BP-DA**, and (**c**) **BP-Br** and **BP-TA**.

In the synthetic route (see Scheme 1), we implemented the previously reported optimized one-pot method [20,21], starting from thioamide to prepare the corresponding amidrazone. Subsequently, the intermediate **1** obtained by condensation of the amidrazone with 3,5-dimethyl-1*H*-pyrrole-2-carbaldehyde was further complexed with boron trifluoride etherate to obtain the BOPAM molecule. This method allows for the facile introduction of substitutions on the boron center, not only avoiding careful control of the harsh reaction conditions employed in previous literature, but also preventing the occurrence of multiple substitutions within the system [22,23,24]. To elucidate the influence of substituents on the photophysical properties, structures were designed and modified based on two sites on the BOPAM skeleton, namely the boron and the carbon-linked aryl group of the amidrazone.

First, two BOPAMs (**BP-Ph** and **BP-Na**) were designed through the introduction of phenyl and naphthyl groups to the boron atom (of the 5-membered ring), respectively. The ligand **1** selectively reacts with diphenylborinic acid or di(2-naphthyl)borinic acid under mild conditions (room temperature) to form monochelated arylboron complex **2** or **3**, which is further complexed with boron trifluoride etherate to obtain the final compound **BP-Ph** or **BP-Na**, respectively (Scheme 1a). Second, a diphenylamine (**DA**) group was introduced into the phenyl ring of the BOPAM skeleton. Starting with 4-(diphenylamino)benzaldehyde, 4-(diphenylamino)-*N*-phenylbenzothioamide was synthesized with a high yield through a three-step reaction sequence (see Scheme S1), followed by our three-step one-pot method to obtain the target product **BP-DA** (Scheme 1b). Finally, another modification involved attaching a bromine atom onto the phenyl group of the BOPAM skeleton to obtain **BP-Br**. Thus, the triphenylamine group (**TA**) was introduced into **BP-Br** through the Suzuki coupling reaction to obtain **BP-TA** (Scheme 1c).

Products were characterized through NMR spectroscopy (^1^H, ^13^C, ^11^B, and ^19^F) and TOF-HRMS, and their molecular geometry was further confirmed by single-crystal X-ray diffraction analysis (SC-XRD) (more details are summarized in the Supplementary Materials). The analysis of their SC-XRD structures is discussed in the next section.

### 2.2. Structural Characterization by SC-XRD

**Figure 2:**
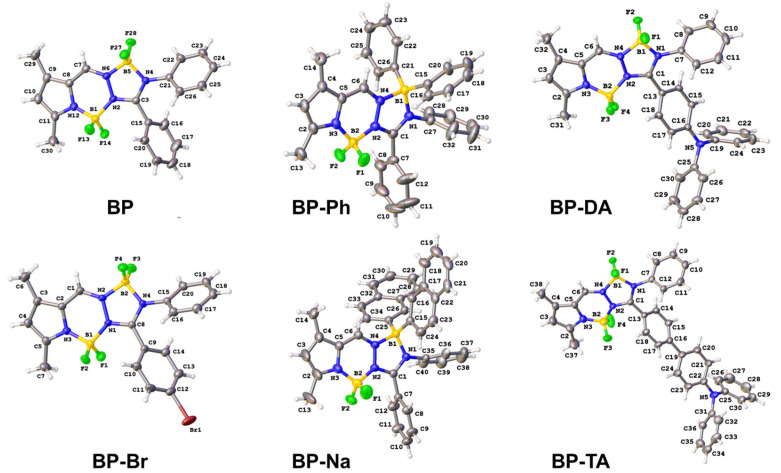
X-ray structures of **BP** [20], **BP-Br**, **BP-Ph**, **BP-Na**, **BP-DA**, and **BP-TA**. Displacement ellipsoids drawn at the 30% probability level.

In this study, the solid state emission properties of BOPAM were rationalized in terms of the single crystal X-ray diffraction (SC-XRD) analyses (Figure 2). Specific intermolecular interactions are discussed below and support the investigation of their emission in a solid state (see Section 2.6).

In the first observation, the central 12-membered ring system was planar with root mean square (r.m.s) deviations from planarity between 0.048 Å (for molecule B in **BP-Na**) and 0.162 Å (for **BP-DA**). **BP-Br** crystallized in the orthorhombic space group *Pbca*, while the parent **BP** crystallized in the monoclinic space group *I*2/*a* [20]. The r.m.s. deviation of fitting the central 12-membered ring of both structures was 0.127 Å (Figure S1a). Both molecules were characterized by an intramolecular C-H⋯F interaction (H22⋯F28 = 2.53 Å in **BP**, H10⋯F2 = 2.45 Å in **BP-Br**). The crystal packing of both molecules showed C-H⋯F interactions for **BP**, and C-H⋯F, C-H⋯π and C-Br⋯π interactions for **BP-Br** (Table S1). Notably, **BP** exhibited an even and uniform arrangement with *head-to-tail* stacking between two (plane ***A***) aromatic system cores (Figure 6b). Upon the introduction of a bromine atom in **BP-Br**, alterations in the orientation of the phenyl rings were observed (refer to Table 1), resulting in a more disordered spatial arrangement (Figure S2a). The phenyl group in plane ***C*** of **BP** demonstrated a greater degree of twisting compared with that of **BP-Br**, whereas the phenyl group in plane ***B*** in **BP** was less twisted than the corresponding group in **BP-Br**, relative to the aromatic core (plane ***A***) (see Table 1).

The spatial arrangement of **BP-Ph** and **BP-Na** in the solid state was predicted to be disordered, requiring a direct observation via SC-XRD analysis. **BP-Ph** crystallized in the monoclinic space group *P*2_1_/*n*, whereas **BP-Na** crystallized in the triclinic space group *P*-1, with two molecules in the asymmetric unit. Both molecules in the asymmetric unit of **BP-Na** differed in the orientation of one of the naphthyl groups (r.m.s. deviation of fitting the central 12-membered ring of both molecules is 0.110 Å, Figure S1b). The substitution of both fluorine atoms on boron by two phenyl rings in **BP-Ph** or two naphthyl groups in **BP-Na** had a major effect on the orientation of the adjacent phenyl groups (in planes ***B*** and ***C***) (Table 1). Only one example of C-H⋯F interaction was observed in the crystal packing of **BP-Ph**, and numerous C-H⋯F and C-H⋯π interactions were present in the crystal packing of **BP-Na** (Table S2). However, there was no specific packing arrangement, such as *herring-bone* motif or *head-to-tail* interactions, and displayed a notably disordered spatial arrangement (Figure S2b,c). This structural disorder suggests that the emission of **BP-Ph** and **BP-Na** in the solid state originates from the intrinsic ^1^LE emission of the BOPAM core or ^1^ICT emission.

Both compounds **BP-DA** and **BP-TA** crystallized in the triclinic space group *P*-1. The extension of the conjugated system did not cause major changes in the dihedral angles between the best plane through the 12-membered ring and the phenyl substituents (Table 1). The extra nitrogen was planar with a deviation from the best plane through three neighboring C atoms of 0.107(2) Å for **BP-DA** and 0.082(4) Å for **BP-TA**. Phenyl rings C13–C18 and C19–C24 in **BP-TA** formed a dihedral angle of 27.41(15)°. An intramolecular C-H⋯F interaction was present (H18⋯F3 = 2.53 Å for **BP-TA** and 2.40 Å for **BP-DA**). Both crystal packings were stabilized by C-H⋯F and C-H⋯π interactions, but for **BP-DA**, B-F⋯π interactions also contributed (Table S3). Notably, both **BP-DA** and **BP-TA** displayed an even and uniform molecular arrangement, characterized by a *head-to-tail* stacking pattern between adjacent triphenylamine groups (see Figure 6c,d). This structural feature facilitates charge transfer processes in the solid state.

### 2.3. UV–Vis Absorption and Emission Properties

The photophysical characteristics of BOPAM were investigated through steady-state spectroscopic measurements. The UV–Vis absorption and emission spectra were recorded in toluene (Tol), tetrahydrofuran (THF), acetonitrile (ACN), and ethanol (EtOH) at a concentration of 10 µM. Complementary to the experimental analyses, their absorption and emission properties were determined with TD-DFT calculations using the PBE0/6-31+G(d) level of theory within the integral equation formalism of the polarizable continuum model (IEFPCM) to model solvent effects in Tol and ACN (see the Section 3 for the full computational details).

**Figure 3:**
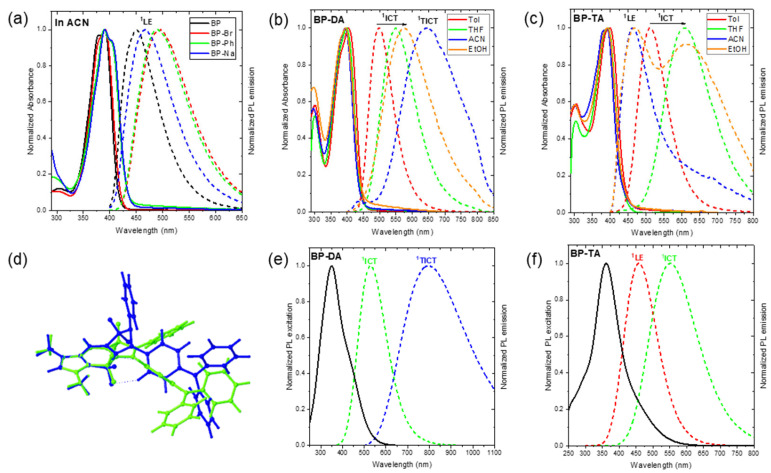
Normalized UV–Vis absorption (solid line) and fluorescence emission (dot line) (λ_ex_ = 390 nm) spectra of (**a**) **BP**, **BP-Br**, **BP-Ph**, and **BP-Na** (10 µM) in acetonitrile (ACN); (**b**) **BP-DA** (10 µM) in toluene (Tol), tetrahydrofuran (THF), (ACN), and ethanol (EtOH); (**c**) **BP-TA** (10 µM) in Tol, THF, ACN, and EtOH; (**d**) overlay of the optimized geometries of ^1^ICT (green) and ^1^TICT (blue) states of **BP-DA**. Calculated absorption (solid line); and emission (dot line) spectra from the ^1^ICT, ^1^LE, and ^1^TICT states of (**e**) **BP-DA** and (**f**) **BP-TA** using TD-PBE0/6-31+G(d) in IEFPCM for ACN.

The absorption spectra of all BOPAM derivatives exhibited their most intense absorption bands at 389–403 nm, characterized by a similar spectral shape and strong absorbance (ε = 35.8–42.5 × 10^3^ cm^−1^ M^−1^) (Figure 3a–c and Figure S3, Table 2). Their absorption maxima demonstrated a relatively weak dependence on solvent polarity, minimal solvatochromic shifts (<10 nm) across Tol, THF, ACN, and EtOH. The absorption spectra of **BP**, **BP-Br**, **BP-Ph**, and **BP-Na** were quite broad (FWHM = 3225–5150 cm^−1^, see Table S4) compared with that of the BODIPY [25,26] nearly structureless band, which showed a red shift from ACN over THF to Tol, and can be correlated with the increase in the polarizability of the solvent [25]. This suggests that the investigated BOPAMs had no or only a very small dipole moment in their ground states. Upon closer inspection, their absorption bands showed a weak shoulder at the blue side (**BP**, **BP-Br**) or the red side (**BP-Ph**, **BP-Na**), which can be attributed to the 0-1 or 0-0 vibronic bands, respectively. This means that while for **BP** and **BP-Br**, the maximum corresponded to the 0-0 vibronic band, it corresponded to the 0-1 transition in **BP-Ph** and **BP-Na**. Furthermore, for **BP-DA** and **BP-TA**, a shoulder with low intensity could be observed between 500 and 600 nm, especially in EtOH. The 0-0 vibronic band of **BP-Ph** and **BP-Na** was red shifted at about 1200 cm^−1^ versus that of **BP**, **BP-Br**, and **BP-TA**.

Notably, the most intense absorption band in **BP**, **BP-Br**, **BP-Ph**, and **BP-Na** was assigned to the S_0_ → S_1_ transition (f > 0.78), predominantly characterized by a highest occupied molecular orbital (HOMO) → lowest unoccupied molecular orbital (LUMO) transition (>97%) of ^1^LE character (Table S7). Conversely, for **BP-DA** and **BP-TA**, the strongest absorption was attributed to the S_0_ → S_2_ transition (f > 0.70), mainly governed by the HOMO-1 → LUMO transition (Table S7) of ^1^LE character. The HOMO and LUMO in **BP**, **BP-Br**, **BP-Ph**, and **BP-Na** as well as the HOMO-1 and LUMO in **BP-DA** and **BP-TA** were primarily localized within the BOPAM core (Figure S4). Furthermore, the associated electronic transitions exhibited highly negative hole-electron distribution indices (t < −1.3) (Figure S5), indicating that the absorption band with a peak around 395 nm of all of the studied BOPAMs corresponded to a purely ^1^LE transition.

Notably, the S_0_ → S_1_ transition in **BP-DA** and **BP-TA** was primarily contributed by the HOMO → LUMO transition (Table S7), with the HOMO residing in the triphenylamine moiety and the LUMO localized within the BOPAM core (Figure S4). The associated electronic transition exhibited highly positive hole-electron distribution indices (t-index = 3.08 and 6.58 Å, for **BP-DA** and **BP-TA**), indicative of an ^1^ICT transition. However, the oscillator strength (*f*) of this transition (0.29–0.43) was notably lower than that of the ^1^LE absorption (0.70–0.91). The ^1^ICT transition could be responsible for the long wavelength shoulder observed for **BP-DA** and **BP-TA**. Additionally, a higher-energy absorption band around 300 nm was observed in **BP** and **BP-Br** (see Figure 3a and Figure S3), which was assigned to the S_0_ → S_3_ transition (see Table S7), exhibiting an ^1^LE character (see Figure S5). In **BP-Ph** and **BP-Na**, the absorption band in this region (see Figure 3a and Figure S3) was attributed to the S_0_ → S_4_ transition (see Table S7), primarily associated with ^1^ICT characteristics (see Figure S5). Meanwhile, **BP-DA** and **BP-TA** (see Figure 3b,c) exhibited an S_0_ → S_4_ transition (see Table S7), which was assigned to the ^1^LE transition (see Figure S5) of the triphenylamine moiety.

### 2.4. The Presence of a Dark State

**BP**, **BP-Br**, **BP-Ph**, and **BP-Na** exhibited a strong unstructured fluorescence emission (Φ_F_ = 45.0–33.5% in Tol) (Figure 3a). The Stokes shift of **BP** and **BP-Br** amounted to 3230 and 3710 cm^−1^ in Tol, while that of **BP-Ph** and **BP-Na** was apparently much larger (5010 cm^−1^ and 4630 cm^−1^, respectively). However, one should take into account that while for **BP** and **BP-Br**, the maximum of the absorption spectrum corresponded to the 0-0 vibronic band, it corresponded to the 0-1 vibronic band for **Bp-Ph** and **BP-Na**. For **BP-Ph** and **BP-Na**, when the shift between the 0-0 vibronic band of the absorption spectrum (shoulder) and the fluorescence maximum was calculated, this quantity was reduced to 3850 and 3320 cm^−1^, respectively, which was of the same magnitude as the Stokes shift observed for **BP** and **BP-Br**.

For **BP**, **BP-Br**, **BP-Ph**, and **BP-Na**, the Stokes shift increased slightly (by 190 to 520 cm^−1^) between Tol and ACN (Figure S3a, Table 2 and Table S5). This increase in the Stokes shift was accompanied by an increase in the FWMH of their corresponding emission spectra. This indicates that the excited state is characterized by a small dipole moment. One should note that in analogy to the earlier investigated BOPAM derivatives [20], the observed Stokes shift was much larger than that found for the BODIPY derivatives [25]. This suggests a significant change in some bond lengths or angles upon excitation, which was also reflected in the larger FWHM of the emission spectra. For **BP**, **BP-Br**, **BP-Ph**, and **BP-Na**, the Φ_F_ decreased by a factor of two to three when increasing the solvent polarity from Tol to ACN. This possibly suggests that a charge transfer state is involved in the internal conversion to the ground state and that its accessibility is determined by the polarity of the solvent (i.e., we recall that while the energy of the ^1^ICT state will be strongly affected by the solvent polarity, this is not the case with the ^1^LE state). The incorporation of a bromine atom into **BP** induced a red-shifted emission (Table 2), which can be attributed to a decrease in the LUMO energy level (Figure S7). The Φ_F_ value of **BP-Br** remained comparable to that of **BP**, suggesting a negligible heavy-atom effect in organic solvents. The introduction of phenyl (**BP-Ph**) and naphthyl (**BP-Na**) substituents at the boron atom resulted in a reduction in the HOMO-LUMO energy gap (Figure S7), thereby leading to a red-shifted emission compared with **BP** in organic solvents (Table 2). Consistent with their S_1_ absorption characteristics, the S_1_ emission of **BP**, **BP-Br**, **BP-Ph**, and **BP-Na** was primarily attributed to a pure ^1^LE transition, as supported by the computed results presented in Table S8 (for emission characteristic) and Figure S7 (for MO) and Figure S8 (for hole-electron analysis).

Conversely, **BP-DA** in Tol already had a larger Stokes shift of 4810 cm^−1^, which was increased in EtOH to 7890 cm^−1^ (Figure 3b). This suggests that the emission occurs from a highly polar excited state. The increase in the Stokes shift (4810–7890 cm^−1^) was accompanied by an increase in the FWHM of the emission band (3380–5000 cm^−1^) and a decrease in the Φ_F_ values (49.1–3.6%) as the solvent polarity increased from Tol to THF and EtOH, displaying the characteristic behavior of an ^1^ICT (Figure 3b and Table 2). This behavior was further corroborated by the computational results, consistent with its assigned S_1_ emission characteristics (i.e., we recall the computed ^1^ICT character of S_1_, see Figure 3 and Figure S7, Table S8). However, in ACN, **BP-DA** exhibited a broad emission band with a peak centered at 644 nm and a significantly low quantum yield (Φ_F_ = 0.3%). This observation further suggests the presence of an additional emissive species in **BP-DA** (e.g., ^1^TICT state).

**Figure 4:**
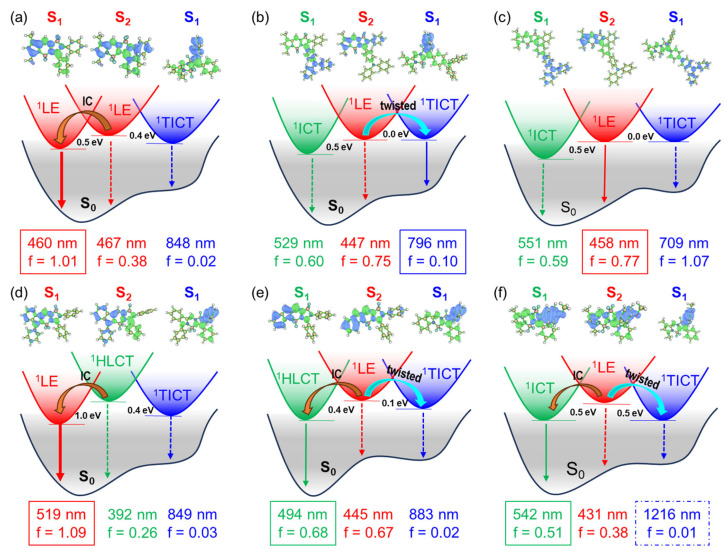
Proposed schematic mechanism for the S_1_ → ^1^TICT transition of (**a**) **BP**, (**b**) **BP-DA**, (**c**) **BP-TA**, (**d**) **aB-BP**, (**e**) **bB-BP**, and (**f**) **BP-OM** illustrated by the hole (blue)-electron (green) distribution and oscillator strength (*f*) and adiabatic energy gaps. Internal conversion (IC).

Previous investigations of BOPAM derivatives [20,21] identified a dark and stable twisted intramolecular charge-transfer (^1^TICT) state/configuration (see Figure 3d) through an integrated computational and experimental photophysical characterization. This ^1^TICT state is known to facilitate non-radiative decay after the S_1_ → ^1^TICT transition because of its typically small oscillator strength and red-shifted emission energy, thus, leading to a significant reduction in fluorescence emission. Accordingly, the optimized geometry of the ^1^TICT state was calculated for compound **BP-DA** (see Figure 3d). Computational results predicted an emission wavelength of 796 nm (in ACN) for the ^1^TICT state of **BP-DA**, which was notably shorter than that of previously reported BOPAM derivatives such as **BP-OM** (1216 nm) and **bB-BP** (883 nm). Moreover, the oscillator strength of the ^1^TICT → S_0_ transition of **BP-DA** (0.10) was significantly larger than that of **BP-OM** (0.01) and **bB-BP** (0.02), suggesting a greater likelihood of observing the ^1^TICT emission experimentally (see Figure 3b,e). Moreover, the oscillator strength of the ^1^TICT state in **BP-DA** (0.1) was markedly lower than that of its ^1^ICT state in Tol (0.3) and ACN (0.6) (Table S8), which was consistent with the reduced Φ_F_ value observed for **BP-DA** in ACN relative to the Tol, THF, and EtOH solvents. Based on these findings, we propose that a broad emission band with a peak centered at 644 nm of **BP-DA** in ACN can be assigned from the weak-emissive ^1^TICT state/configuration (see Figure 4b). This observation further reinforces the existence of non-emissive (dark) or weak-emissive ^1^TICT states in BOPAM derivatives.

Similar to the emission behavior of **BP-DA** in Tol, **BP-TA** exhibited a significantly larger Stokes shift than **BP**, **BP-Br**, **BP-Ph**, or **BP-Na**, which was further increased by 3470 cm^−1^ and 3590 cm^−1^ in THF and EtOH, respectively (see Table S5); a behavior typical for ^1^ICT emissions. This increased Stokes shift was accompanied by an increase in the FWHM of the emission band and a decrease in the FLQY from 62.1% (in Tol) to 51.0% and 1.2% (for the ^1^ICT emission) in THF and EtOH, respectively (Figure 3c). In ACN, the ^1^ICT emission was below the detection limit, although a residual ^1^LE emission was observed (*cfr. infra*). Considering the larger increase in the Stokes shift (or the larger red shift) when going from toluene to THF or EtOH, the ^1^ICT state of **BP-TA** had a larger dipole moment than that of **BP-DA**. Computational analysis further corroborated the presence of the ^1^ICT state in **BP-TA** (see Figure 3f and Figure S7, and Table S8). In a solvent with higher polarity, it was anticipated that the emission peak of **BP-TA** in ACN would occur at wavelengths longer than 608 nm. However, the observed emission of **BP-TA** in ACN was centered at approximately 462 nm (Figure 3c), which was only slightly longer than the emission peak of **BP** in ACN (450 nm). This deviation suggests the presence of a higher-lying excited state (see Figure 4c), indicative of an anti-Kasha emission with an FLQY of 0.4% [27,28]. This anti-Kasha emission can be attributed to the initially populated S_2_ state, which has a ^1^LE character, and was further confirmed by the computational results in Table S8 and Figure S10f. The similarity of the main absorption band and its oscillator strength of, on the one hand, **BP-TA**, and on the other hand, **BP**, **BP-Br**, **BP-Ph**, and **BP-Na**, suggests that this main absorption band must be attributed to a transition of ^1^LE character. One should note that in EtOH, besides the main emission maximum at 610 nm, a weaker band (PLQY of about 0.4%) at 468 nm was also observed. In Tol and THF, this weak anti-Kasha emission was hidden by the much stronger and less red-shifted ^1^ICT emission.

### 2.5. Revisiting the Excited State Decay of BOPAMs

In this study, we have demonstrated the presence of a ^1^TICT state in **BP-DA**. This behavior is reminiscent of previously reported systems such as **BP-OM** (compound **5i** in ref. [20]) and **bB-BP** (compound **5** in ref. [21]). In the former, the S_1_ → ^1^TICT transition in **BP-OM** was found to occur when the energy gap between these states was negative (see Figure 3c in Ref. [20]). In the latter, the smaller energy gap of **bB-BP** compared with **aB-BP** facilitated the possible S_1_ → ^1^TICT transition in **bB-BP** (see Figure 5 in Ref. [21]). Among the series of newly investigated compounds, several (e.g., **BP-Br**, **BP-Ph**, and **BP-Na**) conformed to this energy gap-based model.

However, **BP-DA** deviated from this trend. Despite possessing a ^1^TICT state as the lowest singlet excited state (S₁), the calculated S_1_ → ^1^TICT energy gap for **BP-DA** was significantly larger (0.49 eV) than those for **BP**, **BP-Br**, **BP-Ph**, **BP-Na**, and **bB-BP** (ranging from 0.10 to 0.28 eV). Nevertheless, an emission from the ^1^TICT state was still observed in **BP-DA**, indicating that the energy gap criterion does not universally apply across this series of compounds.

To ensure methodological consistency, the excited-state properties of **BP-OM**, **aB-BP**, and **bB-BP** were recalculated using the computational protocol employed in this study. The revised S_1_-^1^TICT energy gap for **BP-OM** remained negative (−0.04 eV), whereas those for **aB-BP** and **bB-BP** were significantly positive (0.52 eV and 0.28 eV, respectively). These findings suggest that the population of the ^1^TICT state in **BP-DA** and potentially other BOPAM derivatives proceeds via a mechanism distinct from a direct S_1_ → ^1^TICT transition.

**Figure 5:**
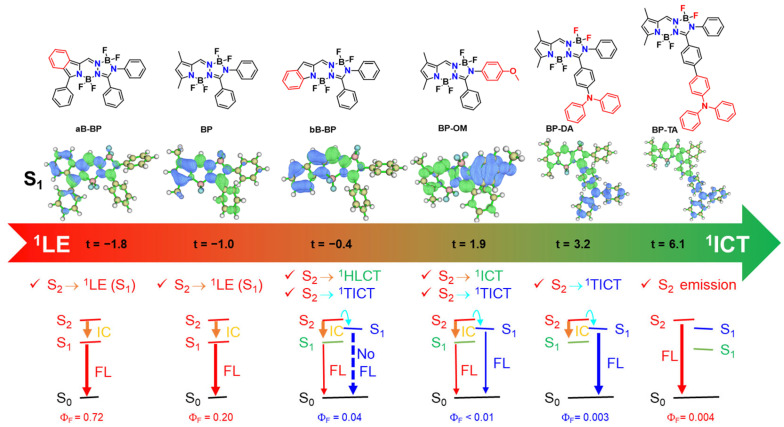
Illustration of the possibility of the transition from S_2_ (^1^LE and ^1^HLCT) to S_1_ (^1^LE, ^1^HLCT, ^1^ICT and ^1^TICT) state of BOPAM. Nature of the states is manifested through the hole (blue)-electron (green) distribution and its t-index (Å). A negative t-index stands for the ^1^LE state while a positive (and larger) t-index correlates with a larger ^1^ICT character.

To elucidate the nature of the S_1_ state in **aB-BP**, **bB-BP**, and **BP-OM**, we conducted an analysis of the hole-electron distribution. The S_1_ state of **aB-BP** exhibited clear ^1^LE characteristics, as both the hole and electron were localized within the same [a]benzo-fused BOPAM core (t = −1.8 Å) (Figure 4d, Figure 5 and Figure S8). Conversely, the S_1_ state of **BP-OM** exhibited distinct ^1^ICT characteristics, with the hole localized in the methoxy phenyl group and the electron in the BOPAM core (t = 1.9 Å) (Figure 4f, Figure 5 and Figure S9). These assignments were further corroborated by the experimental UV–Vis absorption and fluorescence spectra [20,21]. For **bB-BP**, the hole was localized within the benzo fragment, while the electron resided in the BOPAM core (Figure 4e, Figure 5 and Figure S9). The t-index for this state was slightly negative (−0.4 Å), which was significantly higher than the values observed for the ^1^LE states of **BP** (−1.0 Å) and **aB-BP** (−1.8 Å). Additionally, the Stokes shift between the absorption and emission peaks of **bB-BP** (134 nm) was significantly larger than that of **aB-BP** (35 nm) in ACN [21]. The emission peak of **bB-BP** was red shifted from Tol (505 nm) to ACN (531 nm), indicating that the S_1_ state of **bB-BP** is best characterized as a hybridized local and charge-transfer (^1^HLCT) excited state.

Our findings indicate that the population of the ^1^TICT state of BOPAM is suppressed when the S_1_ state of **BP**, **BP-Br**, **BP-Ph**, and **BP-Na** has an ^1^LE character, even in cases where the ^1^LE-^1^TICT energy gap is relatively low (e.g., 0.10–0.18 eV for **BP**, **BP-Br**, **BP-Ph**, and **BP-Na**). However, the ^1^TICT state of **bB-BP**, **BP-OM**, and **BP-DA** is populated when the S_1_ state exhibits ^1^HLCT and ^1^ICT characteristics **(**Figure 4 and Figure 5). These excited states possess charge-transfer features similar to the ^1^TICT state but adopt distinct configurations. The fluorescence emission from the ^1^TICT configuration correlates with the charge-transfer contribution in the S_1_ state. For instance, the fluorescence emission from the ^1^TICT state of **bB-BP** is non-observable [21], whereas the emission from **BP-OM** is ambiguous due to potential overlap with its ^1^ICT emissions [20]. **BP-DA**, which possesses stronger charge-transfer characteristics, exhibited observable ^1^TICT emissions in the fluorescence spectra (see illustration in Figure 5).

Notably, **BP-TA**, which predominantly exhibited ICT characteristics (t-index = 6.1 Å), did not display an observable ^1^TICT emission. However, an emission from the S_2_ state with ^1^LE character was detected in **BP-TA**, indicating a potential role of the S_2_ state in facilitating the population of the ^1^TICT state in BOPAM derivatives. To further investigate this, the second singlet excited states (S_2_) of the BOPAM compounds were optimized using the TD-PBE0/6-31+G(d) level, incorporating the IEFPCM solvent model for ACN. Analysis of the hole-electron distribution revealed that the S_2_ states of most of the studied BOPAM derivatives predominantly exhibited an ^1^LE character, as indicated by the negative t-index values. An exception was the S_2_ state of **aB-BP**, which displayed a slight positive t-index (0.37 Å), suggesting an intramolecular charge-transfer (ICT) character. This state may be classified as an ^1^HLCT state, analogous to the S_1_ state observed in **bB-BP**.

The adiabatic energy of the S₂ state was higher than that of the ^1^TICT state (see Figure 4 and Figure S11), suggesting the potential of an S_2_ → ^1^TICT internal conversion. However, in the cases of **BP**, **BP-Br**, **BP-Ph**, and **BP-Na**, the internal conversion from S_2_ (^1^LE) to S_1_ (^1^LE) dominated, resulting in population of the S_1_ state. Similarly, in **aB-BP**, the primary relaxation pathway involved the internal conversion from S_2_ (^1^HLCT) to S_1_ (^1^LE). In contrast, for **bB-BP**, **BP-OM**, and **BP-DA**, the S_2_ (^1^LE) → S_1_ (^1^TICT) internal conversion competed effectively with the S_2_ (^1^LE) → S_1_ (^1^ICT or ^1^HLCT) internal conversion, leading to the population of the ^1^TICT state. Notably, the ^1^TICT state adopted a twisted geometry in contrast to the planar configuration of the S_2_ and other S_1_ states. In **BP-TA**, the S_2_ state exhibited a purely LE character, as evidenced by the lowest t-index (−1.9 Å) among the BOPAM derivatives studied, indicating a high stability. This enhanced stability renders the S_2_ emission more favorable than the S_2_ → S_1_ (^1^LE, ^1^HLCT, ^1^ICT, and ^1^TICT) transitions [27,28]. Collectively, these findings highlight the significant role of the S_2_ state in facilitating the population of the ^1^TICT state via the S_2_ → ^1^TICT internal conversion. This, in turn, provides insights into the formation and behavior of the weakly emissive or non-emissive ^1^TICT (dark) state in BOPAM derivatives.

### 2.6. Solid State Spectra

**Figure 6:**
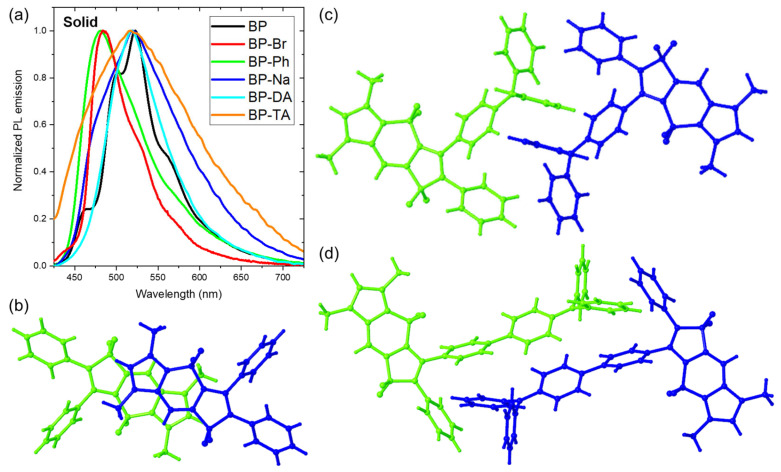
(**a**) Normalized fluorescence emission of BOPAMs in the solid state; partial crystal packing of (**b**) **BP**, (**c**) **BP-DA**, and (**d**) **BP-TA**.

The synthesized BOPAM compounds exhibited a bright emission in the solid state when irradiated with 365 nm light (Figure S12b), which was subsequently quantified via photoluminescence spectroscopy (see results in Figure 6a and Table 2). The emissions displayed a yellow-to-orange hue, consistent with the initial observations of the BOPAMs in our previous study [20]. However, the precise nature and mechanisms underlying their emission behavior in the solid state have yet to be thoroughly elucidated.

Prior to that, their absorption spectra were measured in the solid state. Those of **BP-Ph**, **BP-Na**, **BP-DA** and **BP-TA** consisted of a broad structureless band whose maximum was red shifted by 2000 ± 200 cm^−1^ versus toluene (Figure S12a). For **BP** [20] and **BP-Br**, the red shift of the maximum was less than 400 cm^−1^, but the absorption spectra showed on their red edge a shoulder at 460 and 455 nm, respectively, corresponding to a red shift of 3320 and 3150 cm^−1^ versus toluene. The shoulder and maximum are probably related to vibronic progression. While in the solid state the onset of all absorption spectra was red shifted, suggesting exciton interaction [29,30,31] between neighboring molecules, this red shift was less outspoken for the emission spectra. This can be related to the rigidity of the environment in the solid state or to exciton interaction between neighboring molecules, both leading to less structural relaxation in the excited state [32,33].

The emission spectrum of BOPAM **5a** (**BP)**, which exhibited ^1^LE emissions in both Tol and ACN, consisted of a shoulder and maximum red shifted over 1710 and 2710 cm^−1^, respectively, versus toluene [20]. In contrast, BOPAM **5i** (**BP-OM**), which showed ICT contributions in ACN, experienced a smaller Stokes shift relative to Tol (Δν = 1270 cm^−1^) [20]. As the absorption spectrum (long wavelength shoulder) was red shifted over 3320 cm^−1^ versus toluene, the Stokes shift compared with toluene decreased by 600 and 1600 cm^−1^ for the shoulder and the maximum of the emission spectrum, respectively. In addition, the red shift of the absorption suggests the formation of J-type aggregation, which corresponds to the crystal structure of **BP**, where the A planes of two neighboring molecules were parallel but shifted along their long axis (Figure 6b). **BP** exhibited additional shoulder peaks, which can be rationalized by the presence of strong vibronic coupling. Thus, the emission of **BP** has an ^1^LE character and was red shifted due to the *head-to-tail* parallel arrangement of the A planes at a short distance (3.7 Å), leading to a strong exciton coupling.

The emission spectra of **BP-Br** and **BP-Ph** consisted of a maximum at 484 nm and 481, respectively, compared with toluene of a red shift of 750 cm^−1^ or a blue shift of 380 cm^−1^, respectively. The shoulder and the maximum are possibly part of vibronic progression. This means that compared with toluene, the Stokes shift was reduced by 2400 cm^−1^ (using the long wavelength shoulder of the absorption spectrum) and 2700 cm^−1^ for **BP-Br** and **BP-Ph**, respectively. The observation of **BP-Br** parallels the intrinsic emission of the benzo-fused BOPAM via its brominated derivative in our previous study [21]. Upon the introduction of a bromine atom in **BP-Br**, alterations in the orientation of the phenyl rings were observed (refer to Table 1), resulting in a more disordered spatial arrangement (Figure S2a). This could lead to a weaker exciton interaction, and hence a smaller red shift of the emission between toluene and the solid state. In **BP-Ph**, the exciton interaction was reduced to such an extent that the effect of the reduced excited state relaxation exceeded that of the exciton interaction, resulting in a blue shift of the emission between toluene and the solid state. In addition, the FLQY of **BP-Br** (9.0%) was significantly lower than that of **BP** (32.4%), which can be ascribed to an intermolecular heavy-atom effect. This is consistent with the behavior of the bromide-substituted benzo-fused BOPAM (**5b** in Ref. [21]). **BP-Ph** demonstrated a significant FLQY of 42.7%, suggesting that its emission originates from the intrinsic ^1^LE luminescence of the BOPAM core in the solid state.

The emission spectrum of **BP-Na** in the solid state again resembled that of **BP** and consisted of a shoulder at 502 nm and a maximum at 519 nm, which were red shifted over 570 and 1220 cm^−1^, respectively, versus toluene. The shoulder and the maximum are possibly part of a vibronic progression. As the absorption spectrum was red shifted over 2140 cm^−1^ versus toluene, the Stokes shift was, compared with toluene, decreased by 910 and 1910 cm^−1^ for the shoulder and the maximum of the emission spectrum, respectively. While one would, based on the absence of π–π interactions between neighboring 12 membered rings (A-planes), expect a similar emission spectrum as observed for **BP-Ph**, the emission spectrum of **BP-Na** in the solid state was red shifted by 1220 cm^−1^ (maximum) versus toluene. Furthermore, it was significantly broader than that of **BP**, **BP-Br**, and **BP-Ph**, and its features resembled more than those of **BP-DA** and **BP-TA** (Figure 6a). While **BP** and **BP-Ph** had a FLQY of 32.4% and 42.7%, respectively, that of **BP-Na** was only 15.7%. This suggests that while the emission of **BP**, **BP-Br**, and **BP-Ph** originate in the solid state from a ^1^LE state of the BOPAM, that of **BP-Na** should be attributed to ^1^ICT.

The emission spectra of **BP-DA** and **BP-TA** consisted in the solid state of a broad band with maxima at 518 nm and 517 nm, corresponding to a red shift of 700 cm^−1^ and 190 cm^−1^ compared with toluene. As the absorption spectrum was red shifted by 1830 cm^−1^ and 2140 cm^−1^, respectively, the Stokes shift was again reduced by 1130 cm^−1^ and 1930 cm^−1^. The broad emission bands of both **BP-DA** and **BP-TA**, accompanied by a relatively low FLQY of 11.2% and 6.4%, respectively, suggest that their emission in the solid state is also primarily influenced by charge-transfer processes. The *head-to-tail* stacking pattern between adjacent triphenylamine groups of **BP-DA** and **BP-TA** (Figure 6c,d) will, besides the intramolecular charge transfer occurring in solution, facilitate intermolecular charge transfer processes in the solid state. It is noteworthy that **BP-TA** exhibited a broader emission band and a lower FLQY compared with **BP-DA**, suggesting a more pronounced charge-transfer process in its emission.

## 3. Materials and Methods

### 3.1. General Synthesis of BOPAM

The synthesis of 4-(diphenylamino)-*N*-phenylbenzothioamide is specifically provided in the Supplementary Material File. 4-Bromo-*N*-phenylbenzothioamide, 4-(diphenylamino)-*N*-phenylbenzothioamide, diphenylborinic acid, and di(2-naphthyl)borinic acid were obtained according to the previous literature, and the other synthetic steps are similar to those described in previously reported works [34,35,36].

Compound **1**: *N*-phenylbenzothioamide (2.07 mmol, 441 mg) was dissolved in 20 mL of anhydrous ethanol and stirred at room temperature. Subsequently, hydrazine monohydrate (0.34 mL) was added to the reaction mixture, and the mixture was heated at reflux at 78 °C for 15 min. The reaction progress was monitored using thin-layer chromatography (TLC) until the starting material was fully converted. After cooling to room temperature, the organic phase was extracted three times with ethyl acetate and washed with deionized water. The organic phase was dried over anhydrous sodium sulfate to remove residual moisture, then the solvent was removed using a rotary evaporator to obtain the intermediate for direct use in the next step. 3,5-Dimethyl-2-formylpyrrole (1.72 mmol, 212 mg) and 20 mL anhydrous ethanol were added to the flask, and the mixture heated at reflux after adding 4 drops of glacial acetic acid. The reflux was continued at 78 °C, while monitoring the reaction by TLC until the 3,5-dimethyl-2-formylpyrrole had been completely consumed. The solvent was evaporated under reduced pressure to proceed to the next step.

Compound **2**: Ligand **1** was dissolved in 15 mL of dry dichloromethane (DCM), and diphenylborinic acid (0.8 eq, 1.72 mmol, 313 mg) was added to the solution. The reaction mixture was stirred overnight under a nitrogen atmosphere at room temperature. The solvent was evaporated under reduced pressure to obtain the crude product.

**BP-Ph**: Compound **2** was dissolved in 15 mL of dry toluene, and triethylamine (14 eq, 4 mL) was added. The reaction mixture was stirred at room temperature for 15 min and cooled to 0 °C. Boron trifluoride diethyl etherate (BF_3_·OEt_2_, 16 mmol, 4 mL) was slowly added under nitrogen protection. Then, the mixture was heated at 110 °C overnight. After cooling to room temperature, the reaction was quenched with deionized water, the organic phase was extracted three times with DCM and washed with deionized water. The organic layers were combined, dried over anhydrous sodium sulfate, and concentrated under reduced pressure. The crude product was purified by silica gel column chromatography (eluent: petroleum ether/DCM = 1:1) and recrystallized with a DCM-pentane solvent system to obtain **BP-Ph** in 20% yield, mp: 235–237 °C. ^1^H NMR (400 MHz, CDCl_3_) δ 7.52 (d, *J* = 7.3 Hz, 1H), 7.48–7.28 (m, 3H), 7.04–6.89 (m, 1H), 6.58 (d, *J* = 7.9 Hz, 1H), 6.06 (s, 1H), 2.37 (s, 2H), 2.14 (s, 2H).^13^C NMR (101 MHz, CDCl_3_) δ 163.41, 145.79, 138.36, 133.98, 133.56, 132.32, 131.04, 129.77, 128.44, 128.00, 127.94, 127.85, 127.45, 126.97, 126.29, 122.76, 116.65, 14.02, 11.04.^11^B NMR (128 MHz, CDCl_3_) δ 5.42, 1.21.^19^F NMR (376 MHz, CDCl_3_) δ −132.01, −132.10. HRMS (ESI, [M + H]^+^) for C_32_H_28_B_2_F_2_N_4_; calcd. 529.2541, found: 529.2573.

Compound **3**: Ligand **1** was dissolved in 15 mL of dry dichloromethane (DCM), and di(2-naphthyl)borinic acid (0.8 eq) was added to the solution. The reaction mixture was stirred overnight under a nitrogen atmosphere at room temperature. The solvent was evaporated under reduced pressure to obtain the crude product.

**BP-Na**: Compound **3** was dissolved in 15 mL of dry toluene, and triethylamine (14 eq) was added. The reaction mixture was stirred at room temperature for 15 min and cooled to 0 °C. Boron trifluoride diethyl etherate (16 mmol) was slowly added under nitrogen protection. Then, the mixture was heated at 110 °C overnight. After cooling to room temperature, the reaction was quenched with deionized water, the organic phase was extracted three times with DCM, and washed with deionized water. The organic layers were combined, dried over anhydrous sodium sulfate, and concentrated under reduced pressure. The crude product was purified by silica gel column chromatography (eluent: petroleum ether/DCM = 1:1) and recrystallized with a DCM-pentane solvent system to obtain **BP-Na** in 33% yield, mp: 301–303 °C. ^1^H NMR (600 MHz, CDCl_3_) δ 7.91 (s, 2H), 7.86 (d, *J* = 7.7 Hz, 2H), 7.82 (d, *J* = 8.3 Hz, 2H), 7.80 (d, *J* = 7.7 Hz, 2H), 7.56 (d, *J* = 8.1 Hz, 4H), 7.51–7.45 (m, 4H), 7.40 (t, *J* = 7.5 Hz, 1H), 7.37 (s, 1H), 7.35 (t, *J* = 7.5 Hz, 2H), 6.98 (t, *J* = 7.4 Hz, 1H), 6.90 (t, *J* = 7.8 Hz, 2H), 6.60 (d, *J* = 7.3 Hz, 2H), 6.07 (s, 1H), 2.40 (s, 3H), 2.10 (s, 3H); ^13^C NMR (151 MHz, CDCl_3_) δ 163.52, 146.10, 142.63, 138.37, 134.36, 133.48, 133.22, 133.18, 132.59, 131.25, 131.13, 129.79, 128.55, 128.44, 128.07, 127.86, 127.71, 127.15, 127.07, 126.26, 125.87, 125.69, 122.86, 116.84, 14.08, 11.08. ^11^B NMR (128 MHz, CDCl_3_) δ 5.95, 1.38. ^19^F NMR (376 MHz, CDCl_3_) δ −131.82 (d, *J* = 42.0 Hz). HRMS (ESI, [M + Na]^+^) for C_40_H_32_B_2_F_4_N_4_; calcd. 651.2674, found: 651.2659.

**BP-DA**: **4-(Diphenylamino)-***N***-phenylbenzothioamide** (1.31 mmol, 500 mg) was dissolved in 18 mL of anhydrous ethanol and stirred at room temperature. Subsequently, hydrazine monohydrate (0.2 mL) was added to the reaction mixture, and the mixture was heated at reflux at 78 °C for 15 min. The reaction progress was monitored using TLC until the starting material was fully converted. After cooling to room temperature, the organic phase was extracted three times with ethyl acetate and washed with deionized water. The organic phase was dried over anhydrous sodium sulfate to remove the residual moisture, then the solvent was removed using a rotary evaporator to obtain the intermediate. 3,5-Dimethyl-2-formylpyrrole (1.09 mmol, 134 mg) and anhydrous ethanol (15 mL) were added to the flask, and the mixture heated at reflux after adding 3 drops of glacial acetic acid. The reflux was continued at 78 °C, while monitoring the reaction by TLC until the 3,5-dimethyl-2-formylpyrrole had been completely consumed. The solvent was evaporated under reduced pressure to proceed to the next step. Triethylamine (14 eq, 2.2 mL) and dry toluene (14 mL) were added to the intermediate bottle under nitrogen protection. The reaction mixture was stirred at room temperature for 15 min and cooled to 0 °C. BF_3_·OEt_2_ (16 mmol, 2.2 mL) was slowly added under nitrogen protection. Then, the mixture was heated at 110 °C overnight. After cooling to room temperature, the reaction was quenched with deionized water, and the organic phase was extracted three times with DCM and washed with deionized water. The organic layers were combined, dried over anhydrous sodium sulfate, and concentrated under reduced pressure. The crude product was purified by silica gel column chromatography (eluent: petroleum ether/DCM = 1:1) and recrystallized with a DCM-pentane solvent system to obtain **BP-DA** in 27% yield, mp: 260–262 °C. ^1^H NMR (400 MHz, DMSO) δ 8.72 (s, 1H), 7.40–7.31 (m, 4H), 7.28 (t, *J* = 7.3 Hz, 1H), 7.16 (t, *J* = 7.4 Hz, 1H), 7.10 (d, *J* = 7.0 Hz, 1H), 7.05 (d, *J* = 7.5 Hz, 2H), 6.72 (d, *J* = 8.9 Hz, 2H), 6.30 (s, 1H), 2.35 (s, 2H), 2.30 (s, 3H). ^13^C NMR (101 MHz, CDCl_3_) δ 163.14, 150.54, 150.17, 146.34, 138.62, 137.07, 131.68, 131.03, 129.59, 129.02, 127.04, 126.37, 126.05, 124.61, 122.98, 119.05, 118.27, 116.23, 14.22, 10.97.^11^B NMR (128 MHz, CDCl_3_) δ 3.06, 0.86. ^19^F NMR (376 MHz, CDCl_3_) δ −132.13, −132.23, −145.13, −145.22. HRMS (ESI, [M + H]^+^) for C_32_H_27_B_2_F_4_N_5_; calcd. 580.2461, found: 580.2495.

**BP-Br**: **4-Bromo-***N***-phenylbenzothioamide** (1.2 mmol, 292mg) was dissolved in 10 mL of anhydrous ethanol and stirred at room temperature. Subsequently, hydrazine monohydrate (0.2 mL) was added to the reaction mixture and the mixture was heated at reflux at 78 °C for 15 min. The reaction progress was monitored using TLC until the starting material was fully converted. After cooling to room temperature, the organic phase was extracted three times with ethyl acetate and washed with deionized water. The organic phase was dried over anhydrous sodium sulfate to remove the residual moisture, then the solvent was removed using a rotary evaporator to obtain the intermediate. 3,5-Dimethyl-2-formylpyrrole (1 mmol, 123 mg) and anhydrous ethanol (10 mL) were added to the intermediate, and the mixture heated at reflux after adding 1 drop of glacial acetic acid. The reflux was continued at 78 °C, while monitoring the reaction by TLC until the 3,5-dimethyl-2-formylpyrrole had been completely consumed. The solvent was evaporated under reduced pressure to proceed to the next step. Triethylamine (14 eq, 2 mL) and dry toluene (10 mL) were added to the intermediate bottle under nitrogen protection. The reaction mixture was stirred at room temperature for 15 min and cooled to 0 °C. BF_3_·OEt_2_ (16 mmol, 2 mL) was slowly added under nitrogen protection. Then, the mixture was heated at 110 °C overnight. After cooling to room temperature, the reaction was quenched with deionized water, and the organic phase was extracted three times with DCM and washed with deionized water. The organic layers were combined, dried over anhydrous sodium sulfate, and concentrated under reduced pressure. The crude product was purified by silica gel column chromatography (eluent: petroleum ether/DCM = 1:1) and recrystallized with a DCM-pentane solvent system to obtain the final product **BP-Br** in 35% yield, mp: 295–297 °C. ^1^H NMR (400 MHz, CDCl_3_) δ 7.79 (s, 1H), 7.53–7.46 (m, 2H), 7.36 (d, *J* = 8.6 Hz, 2H), 7.27–7.17 (m, 3H), 7.03 (dd, *J* = 8.0, 1.7 Hz, 2H), 6.14 (s, 1H), 2.35 (s, 3H), 2.30 (s, 3H); ^13^C NMR (101 MHz, CDCl_3_) δ 162.02, 151.09, 139.64, 136.22, 132.06, 131.73, 131.23 (t, *J* = 2.3 Hz), 129.39, 127.67, 126.63, 126.47, 124.22, 123.19, 118.68, 14.28 (d, *J* = 1.9 Hz), 11.08; ^11^B NMR (128 MHz, CDCl_3_) δ 3.05 (t, *J* = 25.3 Hz), 0.72 (t, *J* = 29.7 Hz); ^19^F NMR (376 MHz, CDCl_3_) δ −132.07 (dd, *J* = 53.1, 19.6 Hz), −144.72 (dd, *J* = 49.2, 21.4 Hz). HRMS (ESI, [M − F − H]^+^) for C_20_H_17_B_2_F_4_N_4_Br; calcd. 471.0780, found: 471.0776.

**BP-TA**: **BP-Br** (0.2 mmol, 1 eq), (4-(diphenylamino)phenyl)boronic acid (1.3 eq, 0.26 mmol), Tetrakis(triphenylphosphine)palladium(0) (Pd(PPh_3_)_4_, 0.5 eq, 0.1 mmol), and potassium carbonate (3 eq, 0.6 mmol) were added in a dry round-bottom flask. The mixture was dissolved in dioxane that had been bubbled with nitrogen. The reaction mixture was heated at reflux under a nitrogen atmosphere for 12 h. After cooling to room temperature, the reaction was quenched with deionized water, and the organic phase was extracted three times with DCM and washed with deionized water. The organic layers were combined, dried over anhydrous sodium sulfate, and concentrated under reduced pressure to obtain the crude product. The crude product was purified by silica gel column chromatography (eluent: petroleum ether/DCM = 1:1) and recrystallized with a DCM-pentane solvent system to obtain **BP-TA** in 52% yield, mp: 268–270 °C. ^1^H NMR (400 MHz, CDCl_3_) δ 7.81 (s, 1H), 7.57–7.50 (m, 4H), 7.44 (d, *J* = 8.7 Hz, 2H), 7.29–7.24 (m, 4H), 7.21–7.16 (m, 2H), 7.14–7.01 (m, 11H), 6.14 (s, 1H), 2.36 (s, 3H), 2.31 (s, 3H); ^13^C NMR (101 MHz, CDCl_3_) δ 163.09, 150.69, 148.20, 147.53, 143.56, 139.17, 136.65, 132.94, 131.96, 130.26, 129.48, 129.23, 127.93, 127.36, 126.45, 126.07, 124.82, 123.45, 123.43, 123.33, 123.15, 118.51, 14.28, 11.07; ^11^B NMR (128 MHz, CDCl_3_) δ 3.24 (t, *J* = 26.5 Hz), 0.84; ^19^F NMR (376 MHz, CDCl_3_) δ −132.10 (d, *J* = 46.2 Hz), −144.87 (d, *J* = 35.1 Hz). HRMS (ESI, [M + H]^+^) for C_38_H_31_B_2_F_4_N_5_; calcd. 656.2774, found: 656.2790.

### 3.2. Computational Details

The geometries of BOPAMs in the ground state (S_0_) and excited states (S_1_ and S_2_) were optimized using density functional theory (DFT) [37] and time-dependent density functional theory (TD-DFT) [38], respectively, without imaginary frequency. All calculations were performed using the PBE0 [39]/6-31+G(d) [40] level of theory with the integral equation formalism polarizable continuum model (IEFPCM) to model the solvent effects in acetonitrile and toluene [41], as implemented in the Gaussian 16 package [42]. Excitation properties were computed using TD-DFT at the optimized S_0_ geometry with the same level of theory and solvent model. The excitation and emission energy, computed by PBE0, exhibited the best correlation with the experimental absorption and emission spectra with low MAD (0.10–0.20 eV) compared with other functionals enabling the accurate calculation of charge transfer [43,44] such as CAM-B3LYP [45] (MAD of 0.40–0.43 eV, see Table S6). The latter functional, while describing the CT states more accurately, deteriorated the description of the excited states with predominant LE character, and thus led to a worse agreement with the experimental evidence. Additionally, the hole and electron distributions were analyzed using Multiwfn [46,47].

The total magnitude of the charge transfer length was quantified by the *D*-index [47], defined as:

(1) $$D\equiv \sqrt{\sum_{i=x,y,z}{\left(\left|\int i\rho^{hole}(r)dr-\int i\rho^{electron}(r)dr\right|\right)}^{2}}$$

where ∫iρ(r)dr denotes the *x*/*y*/*z* coordinate of the centroid of the hole or electron distribution. The *H*-index characterizes the average spatial extent of the hole and electron distributions along the charge transfer direction [47] and is given by:

(2) $$H=\left(\left|\sigma_{hole}\right|+\left|\sigma_{electron}\right|\right)/2$$

where σ represents the root-mean-square deviation (RMSD) of the hole or electron density. To further evaluate the degree of spatial separation between the hole and electron along the charge transfer direction, the t-index [47] was introduced:

(3) $$t=D-\left|H\cdot {\vec{u}}_{CT}\right|$$

where ${\vec{u}}_{CT}$is the unit vector along the charge transfer direction, which can be determined from the centroids of the hole and electron distributions. A clearly positive *t*-index indicates significant spatial separation between the hole and electron distributions, corresponding to a strong ICT character. Conversely, a negative *t*-index suggests an LE state, with minimal charge separation.

## 4. Conclusions

We synthesized a series of novel BOPAM derivatives and conducted a comprehensive investigation of their photophysical properties in both the solid state and organic solvents. SC-XRD analysis revealed that the intrinsic ^1^LE emission of BOPAM in the solid state could be observed in **BP-Br** and **BP-Ph**. In contrast, the fluorescence emission of **BP-Na**, **BP-DA**, and **BP-TA** at longer wavelengths exhibited intermolecular and/or intramolecular charge transfer characteristics. Furthermore, the red-shifted emission of **BP** relative to **BP-Br** was associated with a combination of exciton coupling and a phenyl-twisted configuration.

In Tol, **BP**, **BP-Br**, **BP-Ph**, and **BP-Na** predominantly exhibited characteristic an ^1^LE emission, while **BP-DA** and **BP-TA** displayed a distinct ^1^ICT emission. Notably, **BP-DA** showed weak fluorescence originating from the ^1^TICT configuration, which was facilitated by the S_2_ (^1^LE) → S_1_ (^1^TICT) transition. In contrast, **BP-TA** exhibited emissive behavior from a higher-lying S_2_ state in ACN. Thus, we propose that the S_2_ → ^1^TICT transition is facilitated by conformational reorganization when the S_2_ and S_1_ states possess the appropriately locally excited and charge-transfer characteristics, respectively. Conversely, when both the S_2_ and S_1_ states are purely LE and ICT in nature, the S_2_ → ^1^TICT transition is negligible, and emissions from the higher-lying S_2_ (^1^LE) state become prominent.

In summary, this finding elucidates the fluorescence quenching mechanism of BOPAM by confirming the presence of the S_2_ state, the S_2_→^1^TICT transition, and the dark ^1^TICT state/configuration. This study not only contributes to new molecular designs toward red-shifted emissions, but also provides a deeper understanding of the fluorescence quenching of BOPAM-based fluorophores.

## Figures and Tables

**Table 1:** Dihedral angles (°) between planes ***A***, ***B***, and ***C***in BOPAM dyes **BP** [20], **BP-Br**, **BP-Ph**, **BP-Na**, **BP-DA**, and **BP-TA**.

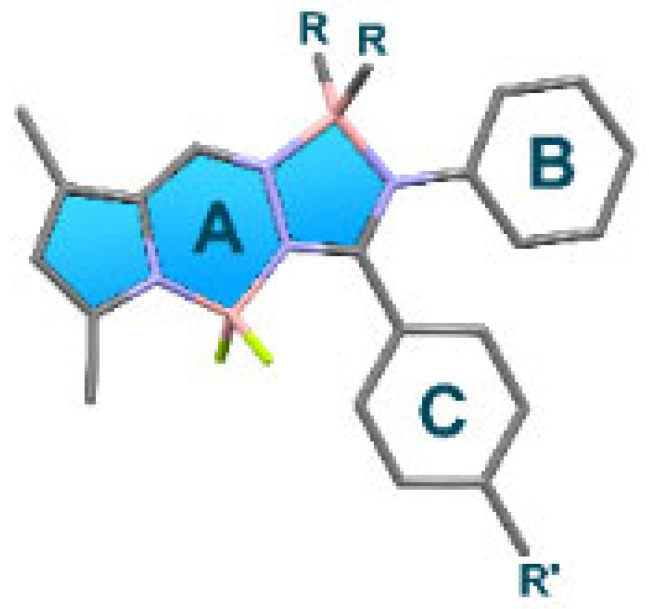	Angle *A*,*B*	Angle *A,C*	Angle *B,C*
**BP**	45.40(8)	74.98(9)	69.07(10)
**BP-Br**	61.35(18)	62.33(18)	58.4(2)
**BP-Ph**	81.34(13)	87.29(16)	68.49(19)
**BP-Na—**molecule 1	74.93(13)	85.91(14)	81.89(17)
**BP-Na—**molecule 2	88.38(12)	73.40(14)	35.52(16)
**BP-DA**	51.05(6)	53.79(8)	56.87(9)
**BP-TA**	60.84(12)	69.13(11)	64.94(14)

**Table 2:** Photophysical properties of **BP**, **BP-Br**, **BP-Ph**, **BP-Na**, **BP-DA**, and **BP-TA** (10 µM) in toluene (Tol), tetrahydrofuran (THF), acetonitrile (ACN), ethanol (EtOH), and the solid state. Maximum absorption wavelength (λ_abs_) (nm); molar absorption coefficient (ε) at maximum absorption wavelength (cm^−1^ M^−1^ 10^−3^); maximum emission wavelength (λ_ems_) (nm); fluorescence emission quantum yield (Φ_F_) (%) [λ_ex_ = 390 nm].

State	BP		BP-Br		BP-Ph		BP-Na		BP-DA		BP-TA	
**Abs.**	**λ_abs_**	**ε**	**λ_abs_**	**ε**	**λ_abs_**	**ε**	**λ_abs_**	**ε**	**λ_abs_**	**ε**	**λ_abs_**	**ε**
Tol	399	39.0	398	40.5	396	40.0	398	38.8	403	34.9	398	41.2
THF	393	36.2	393	37.8	393	37.5	394	41.0	398	36.5	392	36.1
ACN	390	42.5	390	39.3	389	38.5	390	37.3	394	35.8	391	39.0
EtOH	393	40.4	392	39.6	393	39.4	394	39.6	396	43.4	391	36.3
**Ems.**	**λ_ems_**	**Ф_F_**	**λ_ems_**	**Ф_F_**	**λ_ems_**	**Ф_F_**	**λ_ems_**	**Ф_F_**	**λ_ems_**	**Ф_F_**	**λ_ems_**	**Ф_F_**
Tol	458	45.0	467	44.4	494	33.5	488	40.8	500	49.1	512	66.2
THF	454	21.2	469	24.4	496	18.3	492	26.2	548	45.2	608	51.0
ACN	450	19.2	467	19.3	496	11.4	488	15.3	644	**0.3**	462	0.4
EtOH	452	22.1	465	23.9	490	17.4	488	24.6	576	3.6	468; 610	**0.4; 1.2 ^a^**
Solid	523	32.4	484	9.0	481	42.7	519	15.7	518	11.2	517	6.4

^a^ FLQY ^1^LE = 0.4%; FLQY ^1^ICT = 1.2%.

## Data Availability

The original contributions presented in the study are included in the article/Supplementary Materials; further inquiries can be directed to the corresponding authors.

## Supplementary Materials

The following supporting information can be downloaded at: https://www.mdpi.com/article/10.3390/molecules30132673/s1. The Supplementary Materials are available online: general synthesis, structural spectra of compounds, SC-XRD data, computational and photophysical results [48,49,50].
