# Supplementary material for: BOPAM’s Bright and Dark Excited States: Insight from Structural, Photophysical, and Quantum Chemical Investigations
Source: Molecules. 2025 Jun 20;30(13):2673. doi: 10.3390/molecules30132673 (PMC12250827; doi:10.3390/molecules30132673)
Supplement: Supplementary file 1 [file molecules-30-02673-s001.zip › molecules-3642686-supplementary.pdf]

Supporting information of

BOPAM's Bright and Dark Excited States: Insight from Structural, Photophysical and Quantum Chemical Investigations

Kexin Yu,[1,†] Thanh Chung Pham,[1,2,†] Jianjun Huang,[1] Yixuan Li,[2] Luc Van Meervelt,[3] Mark Van der Auweraer,[4] Daniel Escudero,*[2] and Wim Dehaen*[1]

[1] *Sustainable Chemistry for Metals and Molecules, Department of Chemistry, KU Leuven, Celestijnenlaan 200F, 3001 Leuven, Belgium; kexin.yu@kuleuven.be (K.Y.); thanhchung.pham@kuleuven.be (T.C.P.); jianjun.huang@kuleuven.be (J.H.)*

[2] *Quantum Chemistry and Physical Chemistry, Department of Chemistry, KU Leuven, Celestijnenlaan 200F, 3001 Leuven, Belgium; yixuan.li@kuleuven.be*

[3] *Biochemistry, Molecular and Structural Biology, Department of Chemistry, KU Leuven, Celestijnenlaan 200F, 3001 Leuven, Belgium; luc.vanmeervelt@kuleuven.be*

[4] *Molecular Imaging and Photonics, Department of Chemistry, KU Leuven, Celestijnenlaan 200F, 3001 Leuven, Belgium; mark.vanderauweraer@kuleuven.be*

* *Correspondence: daniel.escudero@kuleuven.be (D.E.); wim.dehaen@kuleuven.be (W.D.)*

[†] *The authors contributed equally to this work*

1. Materials and Methods

Reagents, and Solvents: Chemicals received from commercial sources were used without further purification. Reaction dry solvents were dried using a M-Braun SPS-800 system (toluene and tetrahydrofuran).

Instrumentation: NMR spectra were recorded on a Bruker Avance 400 or 600 MHz spectrometer and chemical shifts (δ) are reported part per million (ppm) referenced to tetramethylsilane (TMS, 0.0 ppm), ($CDCl_3$, 7.26 ppm for ^{1}H NMR, 77.16 ppm for ^{13}C NMR; DMSO-d6 for 2.50 ppm for ^{1}H NMR). UV-Vis absorption spectra were recorded on a PerkinElmer Lambda 40 spectrophotometer using blank correction. Fluorescence spectra and excitation spectra were recorded on a HORIBA Jobin Yvon Fluorolog FL3-22 fluorimeter and corrections for the excitation beam intensity, the wavelength dependent sensitivity of the detector and the optical path were applied. Absolute quantum yields were determined with the integrating sphere technique employing the same Horiba spectrofluorometer. A 420 nm long pass filter was used on the detector side to avoid second order scattering effects when collecting the fluorescence spectra. High-resolution mass spectra (HRMS) were obtained using a quadrupole orthogonal acceleration time-of-flight mass spectrometer (Synapt G2 HDMS, Waters, Milford, MA). Samples were infused at 3μL/min and spectra were obtained in positive ionization mode with a resolution of 15000 (FWHM) using leucine enkephalin as lock mass.

2. Synthetic process

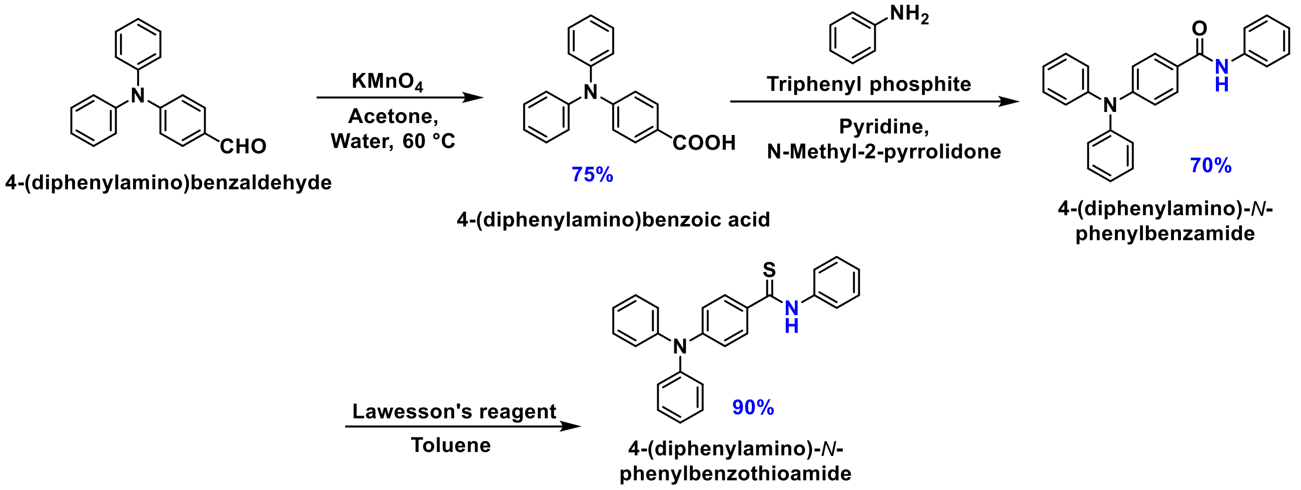

Scheme S1. Synthetic process of **4-(diphenylamino)-*N*-phenylbenzothioamide**.

4-(Diphenylamino)benzoic acid: 4-(Diphenylamino)benzaldehyde (3.66 mmol, 1 g) was dissolved in a mixed solvent of acetone (26 mL) and deionized water (6.4 mL). The reaction solution was stirred at 60°C, and potassium permanganate (4.6 eq, 2.66 g) was gradually added. The reaction was stirred for 24 hours. After the reaction was completed, the reaction mixture was concentrated under reduced

pressure. Then deionized water was added, the filtrate was collected by filtration. Hydrochloric acid (2 M, 200 mL) was added to the filtrate and the resulting white solid was collected by filtration. The solid was dried to obtain 4-(diphenylamino)benzoic acid in 75% yield. ^{1}H NMR (400 MHz, $CDCl_3$) δ 10.75 (s, 1H), 7.91 (d, *J* = 7.5 Hz, 2H), 7.30 (d, *J* = 6.5 Hz, 4H), 7.15 (d, *J* = 6.8 Hz, 6H), 6.98 (d, *J* = 7.5 Hz, 2H).

4-(Diphenylamino)-*N*-phenylbenzamide: 4-(Diphenylamino)benzoic acid (3.8 mmol, 1.1 g), aniline (1.2 eq, 4.56 mmol, 425 mg), triphenyl phosphite (1.3 mL), pyridine (1.3 mL) and N-methyl-2-pyrrolidone (5 mL) were mixed in a round-bottom flask and stirred at 120°C for 3 hours under nitrogen protection. After cooling to room temperature, the organic phase was extracted three times with DCM and washed with deionized water. The organic phase was dried over anhydrous sodium sulfate to remove residual moisture, then the solvent was removed using a rotary evaporator to obtain a crude product. The crude product was purified by column chromatography to obtain desired product in 70% yield. ^{1}H NMR (400 MHz, DMSO) δ 10.06 (s, 1H), 7.89 – 7.82 (m, 2H), 7.80 – 7.71 (m, 2H), 7.47 – 7.26 (m, 6H), 7.22 – 7.04 (m, 7H), 7.01 – 6.93 (m, 2H).

4-(Diphenylamino)-*N*-phenylbenzothioamide: 4-(Diphenylamino)-*N*-phenylbenzamide (1.33 mmol, 486 mg), Lawesson reagent (0.6 eq, 0.8 mmol, 324 mg) and dry toluene (15 mL) were added in a dry reaction bottle, stirred and reacted at 110°C under nitrogen protection for 3 hours. After cooling to room temperature, the solvent was removed by rotary evaporator. The product was purified by silica gel column chromatography (eluent: petroleum ether/DCM = 3:2) to obtain the desired product with a yield of 90%. ^{1}H NMR (400 MHz, DMSO) δ 11.50 (s, 1H), 7.79 (dd, *J* = 13.2, 8.5 Hz, 4H), 7.46 – 7.34 (m, 6H), 7.24 (t, *J* = 7.4 Hz, 1H), 7.19 – 7.09 (m, 6H), 6.91 (d, *J* = 8.7 Hz, 2H).

3. SC-XRD characterization

Single crystals of **BP-Ph** were grown from DCM/MeOH, of **BP-Na** from Chloroform/MeOH, of **BP-DA** from DCM/Hexane, of **BP-TA** from DCM/Cyclohexane, and of **BP-Br** from DCM/n-Pentane.

For the X-ray crystallography, intensity data were collected at 293(2) K on an Agilent SuperNova diffractometer with an Eos CCD detector using MoKα radiation. Using Olex2[48], the structure was solved with the SHELXT[49] structure solution program using intrinsic phasing and refined with the SHELXL[50] refinement package using full-matrix least-squares minimization on F^2. Non-hydrogen atoms were refined anisotropically, and hydrogen atoms were refined in the riding mode with isotropic temperature factors fixed at 1.2 times U_{eq} of the parent atoms (1.5 for the methyl groups). Due to the large thermal motion in **BP-Ph**, the C-C distances in phenyl rings C7-C12 and C27-C32 were restrained to be 1.39(1) Å. The crystal data, data collection, and structure refinement details are summarized in **Table S10**. CCDC 2428797-2428801 contains the supplementary crystallographic data for this paper. These data can be obtained free of charge via http://www.ccdc.cam.ac.uk/conts/retrieving.html (or from the CCDC, 12 Union Road, Cambridge CB2 1EZ, UK; Fax: +44 1223 336033; E-mail: deposit@ccdc.cam.ac.uk).

Table S1. C-H···F, C-H···π and C-Br···π interactions in the crystal packing of **BP** and **BP-Br**.

	D-X (Å)	X···A (Å)	D···A (Å)	D-X···A (°)	Symmetry operation
**BP**					
C7-H7···F27	0.93	2.45	3.368(2)	171	1/2-x, 3/2-y, 3/2-z
C25-H25···F13	0.93	2.50	3.280(3)	142	1-x, 1/2+y, 3/2-z
C29-H29c···F14	0.96	2.49	3.445(3)	171	1/2-x, 1/2-y, 3/2-x
**BP-Br**					
C1-H1···F3	0.93	2.53	3.439(5)	166	1-x, 1-y, 1-z
C19-H19···F2	0.93	2.49	3.421(5)	174	x, 1+y, z
C6-H6B···*Cg*1	0.96	2.94	3.715(6)	138	1-x, -y, 1-z
C17-H17···*Cg*2	0.93	2.94	3.687(5)	139	1-x, 1/2+y, 1/2-z
C12-Br1···*Cg*1	1.897(5)	3.824(2)	4.167(5)	86.62(16)	x, 1/2-y,-1/2+z

*Cg*1 and *Cg*2 are the centroids of rings N3/C2-C5 and B1/N1-N3/C1-C2 in **BP-Br**.

Table S2. C-H···F and C-H···π interactions in the crystal packing of **BP-Ph** and **BP-Na**.

	D-X (Å)	X···A (Å)	D···A (Å)	D-X···A (°)	Symmetry operation
**BP-Ph**					
C10-H10···*Cg*1	0.93	2.94	3.652(6)	135	1/2-x, 1/2+y, 3/2-z
**BP-Na**					
C12-H12···F2	0.93	2.49	3.191(4)	133	2-x, -y, -z
C21-H21···F1	0.93	2.48	3.181(4)	132	1-x, 1-y, -z
C61-H61···F3	0.93	2.45	3.323(4)	157	-x, 2-y, 1-z
C20-H20···*Cg*2	0.93	2.78	3.605(5)	148	x, y, z
C50-H50···*Cg*3	0.93	2.97	3.820(3)	153	1-x, 1-y, 1-z
C59-H59···*Cg*4	0.93	2.96	3.664(5)	133	x, 1+y, z
C68-H68···*Cg*4	0.93	2.69	3.513(4)	148	x, y, z
C78-H78···*Cg*5	0.93	2.87	3.760(4)	160	1-x, 2-y, 1-z

*Cg*1 is the centroid of ring N3/C2-C5 in **BP-Ph**. For **BP-Na**, *Cg*2, *Cg*3, *Cg*4 and *Cg*5 are the centroids of rings N7/C42-C45, C67-C72, C17-C22 and C57-C62, respectively.

Table S3. C-H···F, C-H···π and B-F···π interactions in the crystal packing of **BP-DA** and **BP-TA**.

	D-X (Å)	X···A (Å)	D···A (Å)	D-X···A (°)	Symmetry operation
**BP-DA**					
C6-H6···F2	0.93	2.47	3./342(2)	155	-x, 2-y, -z
C15-H15···F3	0.93	2.51	3.339(2)	148	1+x, y, z
C27-H27···*Cg*1	0.93	2.87	3.755(2)	159	-x, 1-y, 1-z
B2-F3···*Cg*2	1.385(2)	3.9388(15)	4.696(2)	115.04(9)	-1+x, y, z
B2-F4···*Cg*3	1.362(2)	3.0576(14)	3.991(2)	124.60(10)	-x, 1-y, -z
**BP-TA**					
C6-H6···F2	0.93	2.49	3.417(3)	177	-x, -y, -z
C27-H27···*Cg*4	0.93	2.90	3.676(5)	141	1-x, -y, 1-z
C33-H33···*Cg*5	0.93	2.85	3.653(4)	146	-x, 1-y, 1-z

*Cg*1, *Cg*2 and *Cg*3 are the centroids of respectively rings B1/N1/C1/N2/N4, C19-C24, N3/C2-C5 in **BP-DA**. For **BP-TA**, *Cg*4 and *Cg*5 are the centroids of rings N3/C2-C5 and C57-C62, respectively.

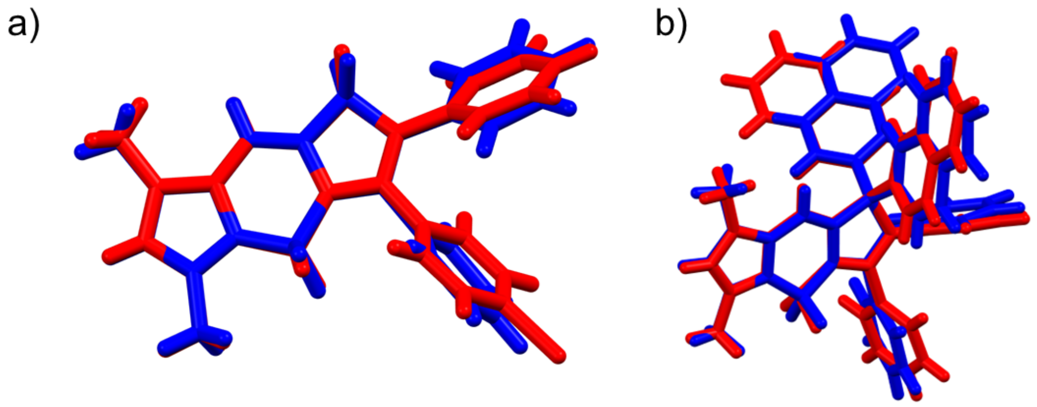

Figure S1. a) Overlay of BOPAM dyes **BP** (blue, ref. [20]) and **BP-Br** (red); b) Overlay of the two molecules present in the asymmetric unit of **BP-Na** (molecule containing atom B1 in blue and atom B4 in red).

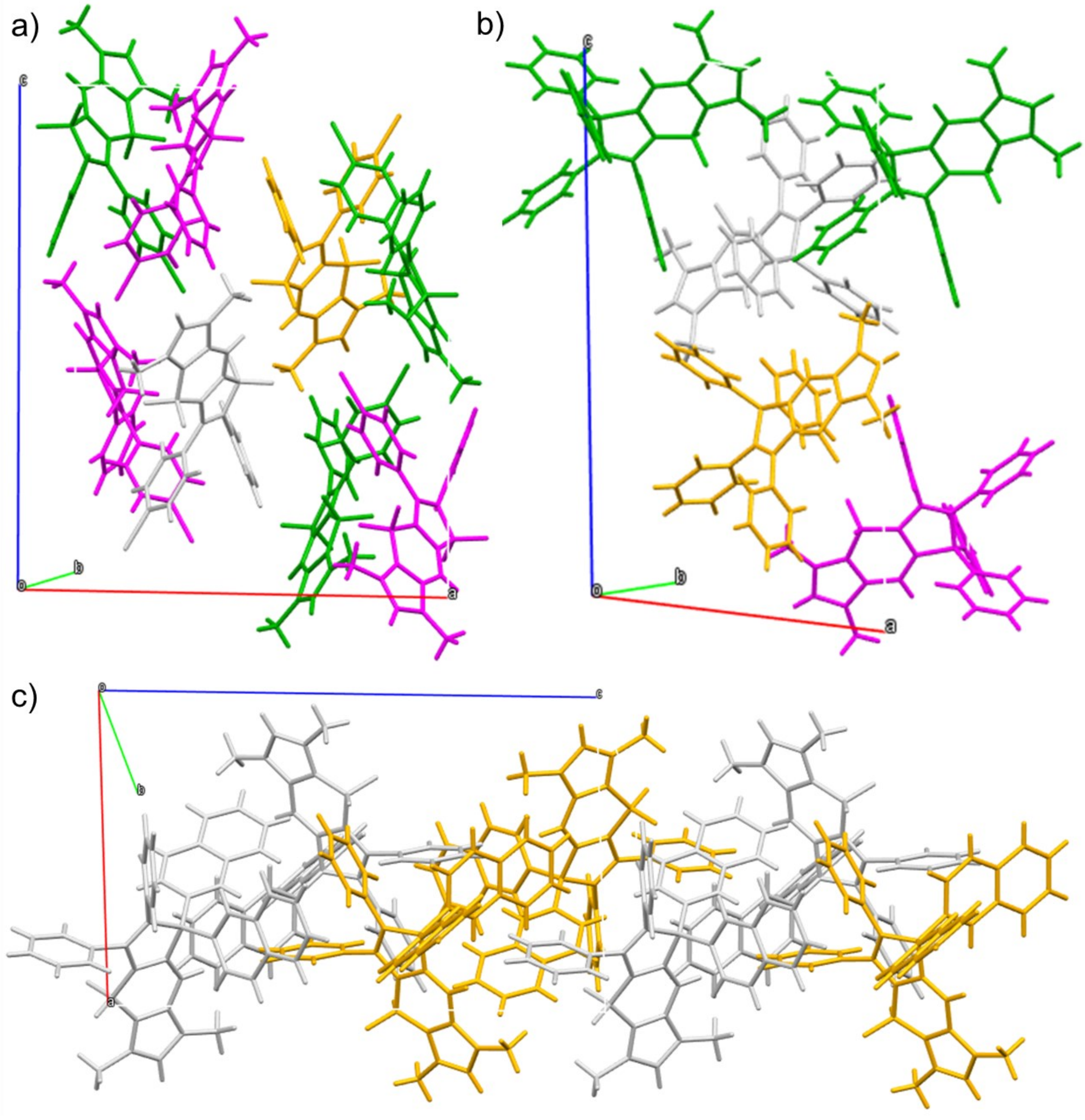

Figure S2. Partial crystal packing of a) **BP-Br**, b) **BP-Ph**, and c) **BP-Na.**

4. Photophysical and computational results

Table S4. Full width at half maximum (FWHM) (cm^{-1}) of absorption (abs) and emission (ems) spectra of BOPAMs in solvents and solid state.

abs/ems	BP	BP-Br	BP-Ph	BP-Na	BP-DA	BP-TA
Tol	3230/3500	3310/3850	3540/4090	3450/4020	4870/3380	4720/3540
THF	3250/3720	3370/4140	3510/4280	3690/4230	4820/3910	4920/4090
ACN	3240/3830	3230/4410	3630/4440	3560/4400	4790/4970	-/4590[a]
EtOH	3480/3710	3360/4150	3610/4240	3680/4270	5150/5000	5920/10000[b]
Solid	-/2450	-/2550	-/3370	-/4330	-/2830	-/5750

a) Emission of the ^{1}LE state, b) overall width of the total spectrum consisting of a ^{1}LE and a ^{1}ICT band

Table S5. Stock shift (cm^{-1}) of BOPAM in solvents.

Sol.	BP	BP-Br	BP-Ph	BP-Na	BP-DA	BP-TA
Tol	3230	3710	5010	4630	4810	5590
THF	3420	4120	5280	5060	6880	9060
ACN	3420	4230	5550	5150	9850	3930[a]
EtOH	3320	4010	5040	4890	7890	4210[a]; 9180[b]

a) Emission of ^{1}LE state, b) emission of ^{1}ICT state

Table S6. Vertical absorption and emission energy (eV) of BOPAMs, computed by TD-DFT in IEFPCM for ACN using 6-31+g(d) basic set and CAM-B3LYP or PBE0 functionals. These energies are compared with experimental (Exp.) ones *via* means absolute deviation (MAD) (in THF*).

	BP	BP-Br	BP-Ph	BP-Na	BP-DA	BP-TA	MAD
**Exp. Abs.**	**3.18**	**3.18**	**3.19**	**3.18**	**3.15**	**3.17**	
CAM-B3LYP	3.66	3.65	3.59	3.55	3.55	3.61	0.43
PBE0	3.45	3.43	3.30	3.29	3.45	3.39	0.20
**Exp. Ems.**	**2.76**	**2.65**	**2.5**	**2.54**	**2.26***	**2.04***	
CAM-B3LYP	2.98	2.95	2.83	2.86	2.80	2.66	0.40
PBE0	2.69	2.62	2.46	2.39	2.34	2.25	0.10

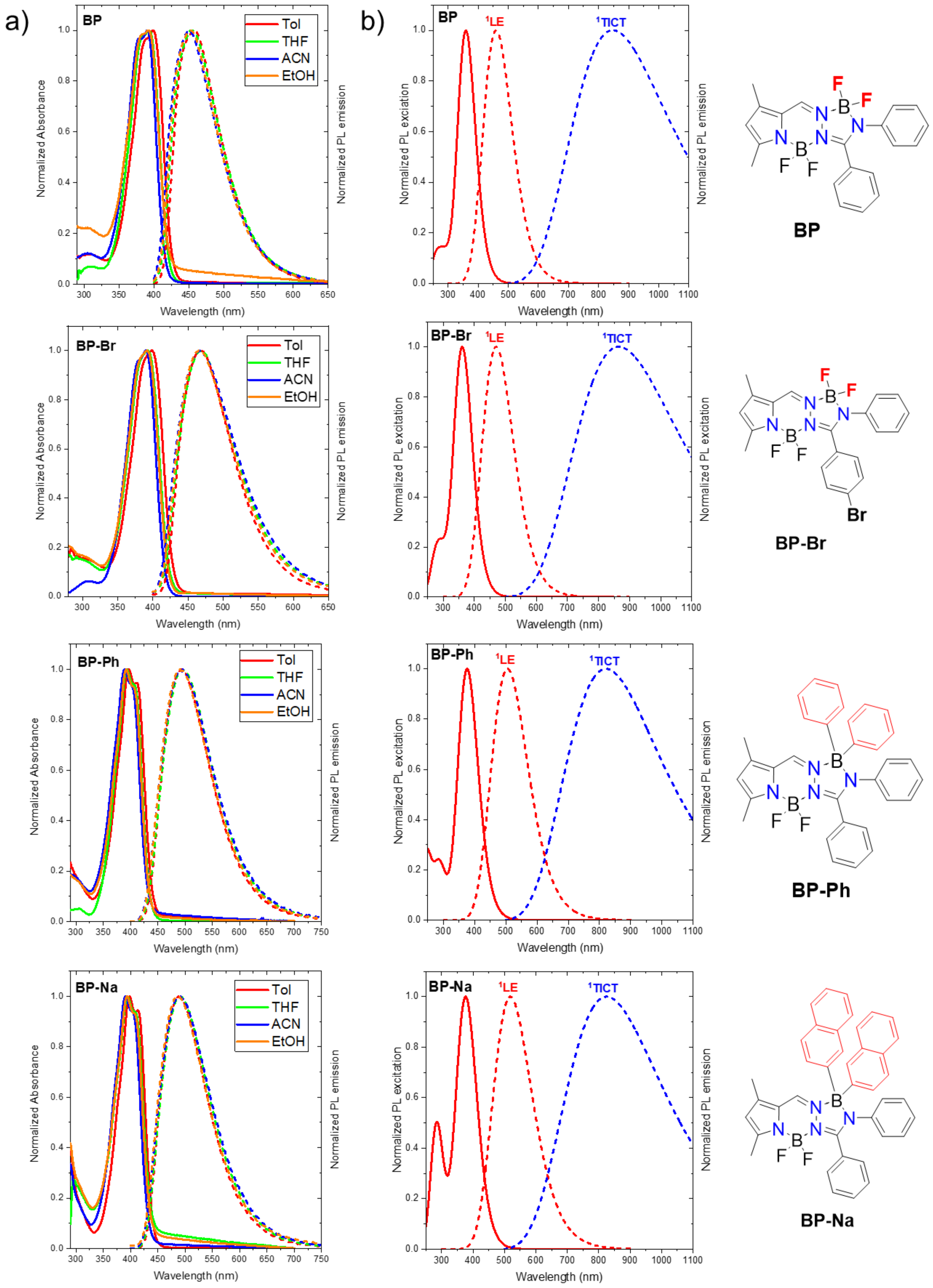

Figure S3. Normalized absorption/excitation (solid line) and emission (dot line) spectra of **BP**, **BP-Br**, **BP-Ph**, and **BP-Na** a) in toluene (Tol), tetrahydrofuran (THF), acetonitrile (ACN), and ethanol (EtOH) (10 μM); b) computed by TD-DFT using PBE0/6-31+g(d) in IEFPCM for ACN.

Table S7. Computed excitation properties of BOPAMs by TD-DFT based on S_0 geometry, which was optimized by DFT method. Both used PBE0/6-31+g(d) level theory in IEFPCM model for toluene and acetonitrile. Vertical energy (E_{vt}) (eV); excitation wavelength (λ_{ex}) (nm); oscillator strength (f); transition (trans); HOMO (H); LUMO (L); the maxima wavelength of experimental (Exp.) of UV-Vis absorption is shown in last column.

		Toluene (Tol)				Acetonitrile (ACN)				Exp.
	State	S_1	S_2	S_3	S_4	S_1	S_2	S_3	S_4	Tol<br>ACN
**BP**	E_{vt}<br>λ_{ex}<br>f	3.38<br>**367**<br>0.88	4.09<br>303<br>0.01	4.21<br>294<br>0.08	4.49<br>276<br>0.01	3.45<br>**359**<br>0.85	4.16<br>298<br>0.00	4.22<br>294<br>0.08	4.54<br>273<br>0.01	**399**<br>**390**
	Trans	H → L<br>97.9%	H-1→L<br>94.8%	H-2→L<br>95.6%	H-3→L<br>61.9%<br>H→L+1<br>32.1%	H→L<br>97.7%	H-1→L<br>78.9%<br>H-2→L<br>18.3%	H-2→L<br>79.6%<br>H-1→L<br>17.9%	H-3→L<br>70.7%<br>H→L+1<br>23.1%	
**BP-Br**	E_{vt}<br>λ_{ex}<br>f	3.35<br>**370**<br>0.88	4.03<br>308<br>0.04	4.19<br>296<br>0.09	4.38<br>283<br>0.03	3.43<br>**362**<br>0.85	4.10<br>302<br>0.02	4.19<br>296<br>0.10	4.42<br>281<br>0.02	**398**<br>**390**
	Trans	H→L<br>97.9%	H-1→L<br>96.5%	H-2→L<br>85.6%<br>H-3→L<br>11.3%	H→L+1<br>58.8%<br>H-3→L<br>32.4%<br>H-2→L<br>5.8%	H→L<br>97.7%	H-1→L<br>92.4%<br>H-2→L<br>5.1%	H-2→L<br>88.2%	H→L+1<br>59.7%<br>H-3→L<br>34.6%	
**BP-Ph**	E_{vt}<br>λ_{ex}<br>f	3.27<br>**379**<br>0.80	4.21<br>295<br>0.06	4.22<br>294<br>0.01	4.25<br>291<br>0.01	3.30<br>**376**<br>0.76	4.20<br>295<br>0.08	4.22<br>294<br>0.00	4.24<br>293<br>0.00	**396**<br>**389**
	Trans	H→L<br>98.1%	H→L+1<br>89.5%<br>H-1→L<br>5.7%	H-2→L<br>73.8%<br>H-1→L<br>21.6%	H-1→L<br>59.3%<br>H-2→L<br>21.3%<br>H-5→L<br>8.3%	H→L<br>98.0%	H→L+1<br>83.8%<br>H-2→L<br>12.6%	H-1→L<br>79.0%<br>H-3→L<br>13.1%<br>H-2→L<br>5.5%	H-2→L<br>72.3%<br>H-1→L<br>9.8%<br>H→L+1<br>9.3%	
**BP-Na**	E_{vt}<br>λ_{ex}<br>f	3.21<br>**386**<br>0.80	3.48<br>357<br>0.02	3.58<br>347<br>0.01	4.03<br>308<br>0.04	3.29<br>**377**<br>0.78	3.54<br>350<br>0.01	3.61<br>344<br>0.02	4.04<br>307<br>0.02	**398**<br>**390**
	Trans	H→L<br>97.9%	H-1→L<br>97.8%	H-2→L<br>98.1%	H→L+1<br>96.0%	H→L<br>97.8%	H-1→L<br>95.4%	H-2→L<br>95.4%	H→L+1<br>95.2%	
**BP-DA**	E_{vt}<br>λ_{ex}<br>f	2.88<br>431<br>0.43	3.40<br>**365**<br>0.71	3.84<br>323<br>0.32	3.88<br>319<br>0.02	2.87<br>432<br>0.37	3.45<br>**359**<br>0.70	3.84<br>323<br>0.34	3.89<br>318<br>0.02	**403**<br>**394**
	Trans	H→L<br>96.2%	H-1→L<br>95.0%	H→L+1<br>91.1%	H→L+2<br>94.5%	H→L<br>96.9%	H-1→L<br>94.0%	H→L+1<br>89.7%	H→L+2<br>92.9%	
**BP-TA**	E_{vt}<br>λ_{ex}<br>f	2.80<br>443<br>0.35	3.34<br>**371**<br>0.88	3.50<br>355<br>0.47	3.78<br>328<br>0.02	2.79<br>445<br>0.29	3.39<br>**365.57**<br>0.91	3.49<br>355<br>0.45	3.79<br>328<br>0.02	**398**<br>**391**
	Trans	H→L<br>95.7%	H-1→L<br>89.6%<br>H→L+1<br>6.5%	H→L+1<br>85.5%<br>H-1→L<br>7.4%	H→L+2<br>92.7%	H→L<br>96.5%	H-1→L<br>65.6%<br>H→L+1<br>29.5%	H→L+1<br>63.5%<br>H-1→L<br>31.5%	H→L+2<br>79.6%<br>H→L+3<br>15.9%	

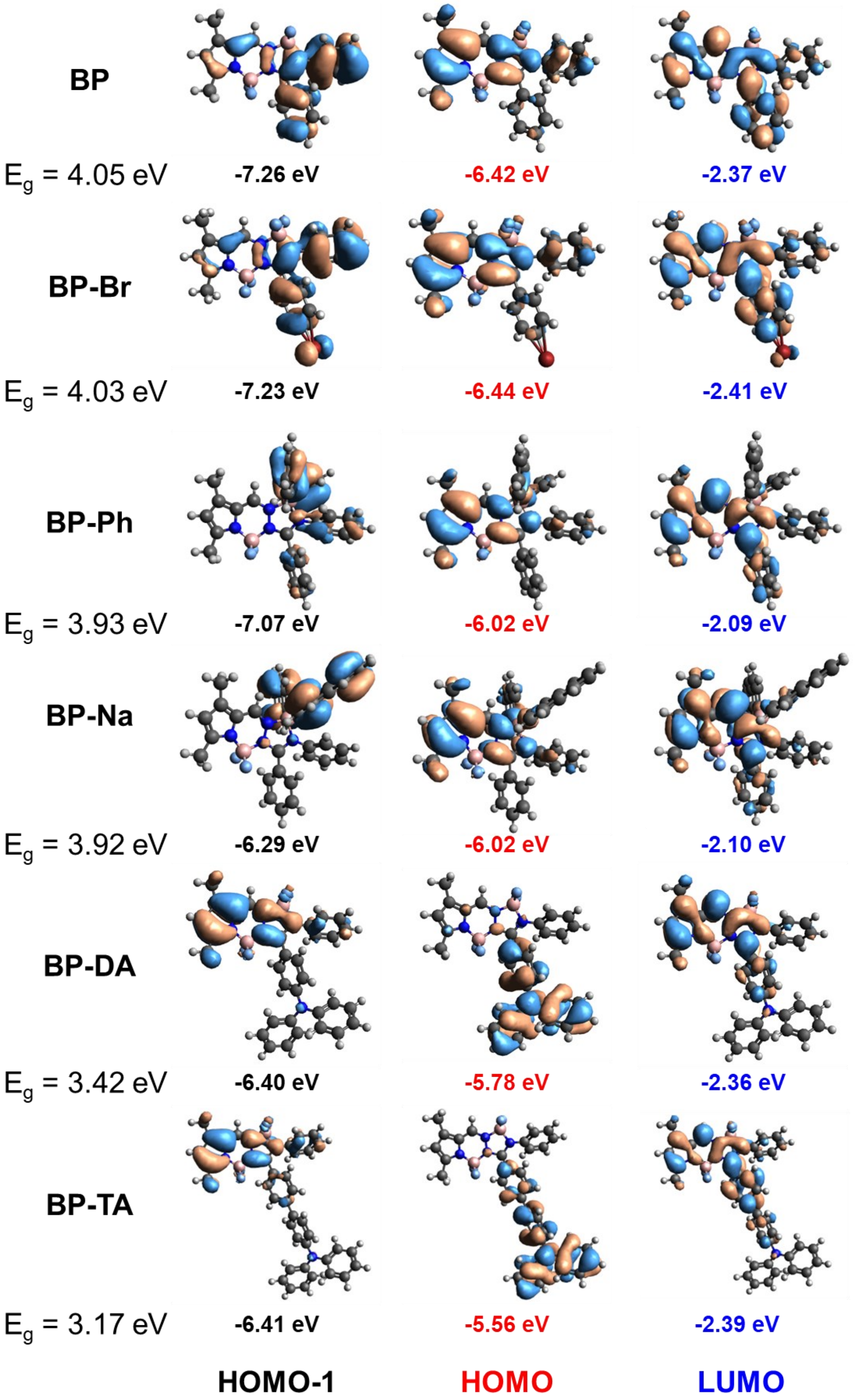

Figure S4. MO images and energy level of BOPAMs at ground state, which was computed by DFT using PBE0/6-31+g(d) in IEFPCM for ACN. HOMO-LUMO energy gap (E_g)

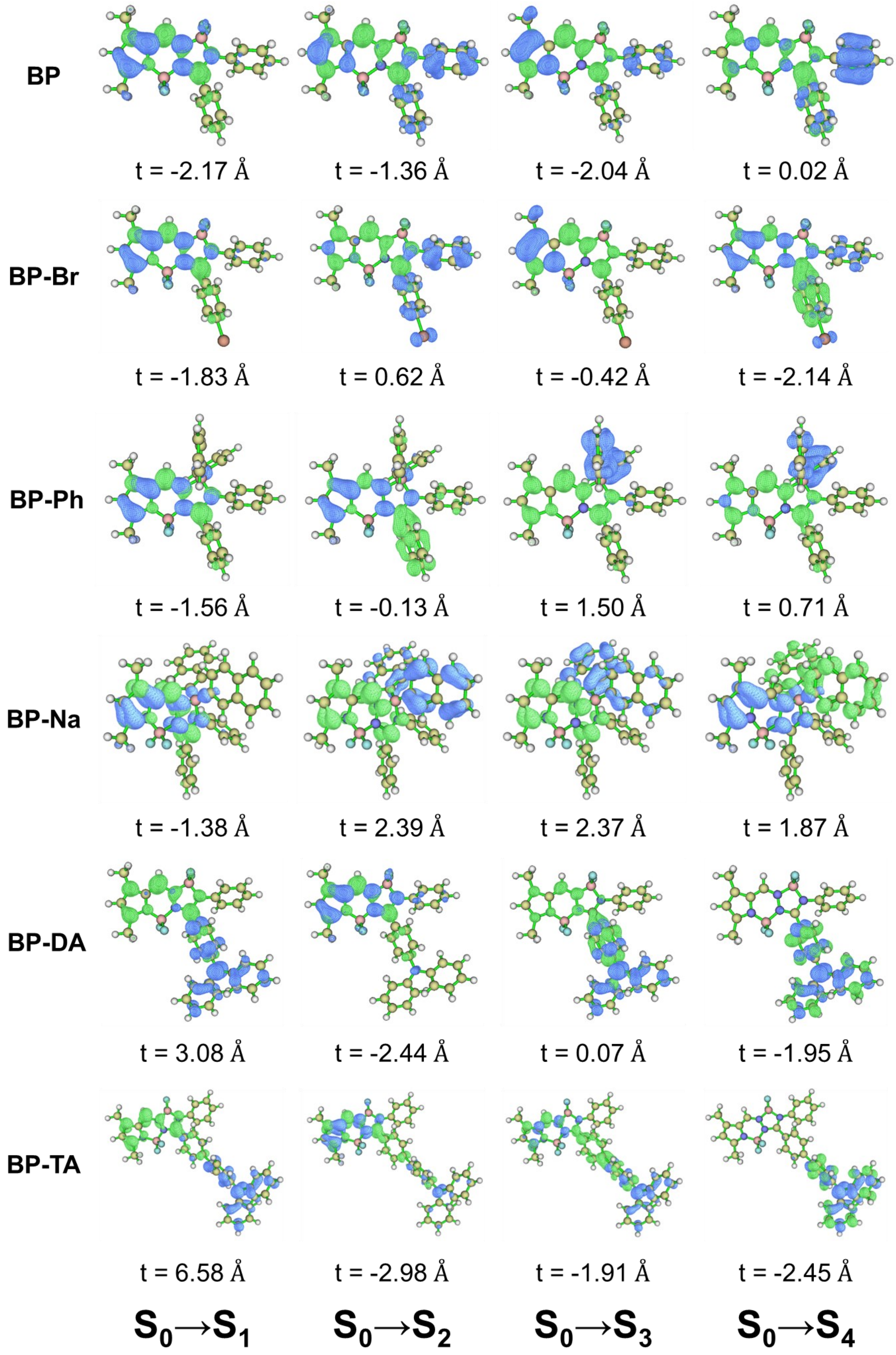

Figure S5. Hole (blue) and electron (green) distribution and its index (t) for excitation transition of BOPAMs, which was computed by TD-DFT using PBE0/6-31+g(d) in IEFPCM for ACN.

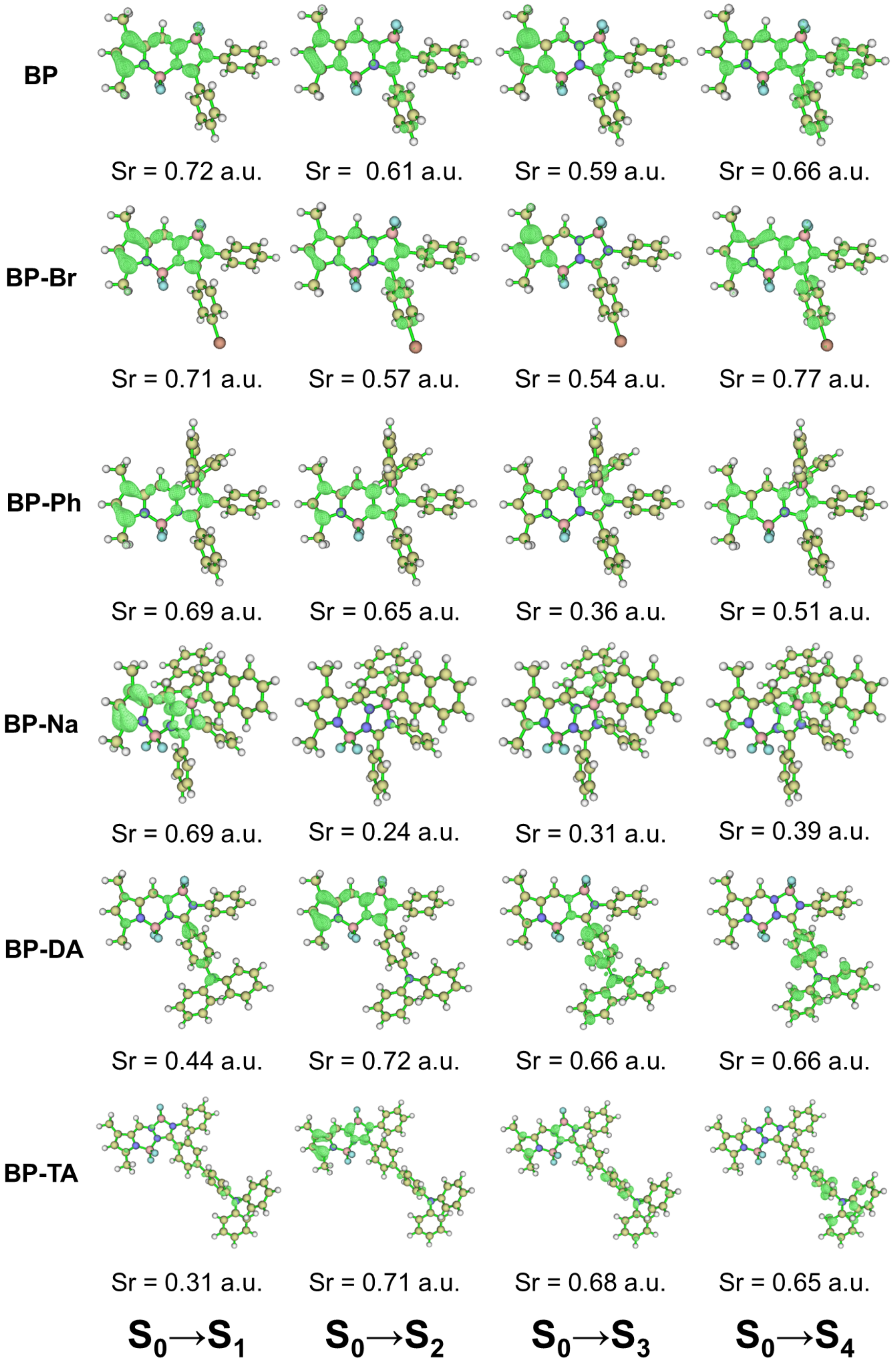

Figure S6. Hole and electron overlap and its index (Sr) for excitation transition of BOPAMs, which was computed by TD-DFT using PBE0/6-31+g(d) in IEFPCM for ACN.

Table S8. Computed emission properties of BOPAMs at [1]LE, [1]ICT, and [1]TICT states (S_1) and [1]LE state (S_2), which was optimized by TD-DFT method using PBE0/6-31+g(d) level theory in IEFPCM model for toluene (Tol) and acetonitrile (ACN). Adiabatic energy (E_{ad}) (eV); vertical energy (E_{vt}) (eV); emission wavelength (λ_{ems}) (nm); oscillator strength (f); the maxima wavelength of experimental (Exp.) fluorescence emission spectra (in THF*).

State	Sol.	E_{ad}	E_{vt}	λ_{ems}	f	transition	Exp.	E_{ad}	E_{vt}	λ_{ems}	f	transition	Exp.
		**BP**						**BP-Br**					
[1]LE	Tol	2.98	2.77	448	0.83	H→L 98.9%	458	2.94	2.70	459	0.80	H→L 99.4%	467
[1]LE	ACN	**2.91**	2.69	460	1.01	H→L 99.0%	450	**2.87**	2.62	471	0.99	H→L 99.1%	467
[1]TICT	ACN	**3.01**	1.46	848	0.02	H→L 98.6%	-	**2.98**	1.43	865	0.03	H→L 98.7%	-
S_2 ([1]LE)	ACN	3.41	2.66	467	0.38	H-1→L 87.1%<br>H→L 11.8%	-	3.35	2.57	483	0.48	H-1→L 84.9%<br>H→L 14.2%	-
		**BP-Ph**						**BP-Na**					
[1]LE	Tol	2.78	2.43	509	0.55	H→L 99.3%	494	2.76	2.43	511	0.54	H→L 99.3%	488
[1]LE	ACN	**2.74**	2.46	505	0.79	H→L 99.2%	496	**2.72**	2.39	519	0.79	H→L 99.0%	488
[1]TICT	ACN	**2.92**	1.51	821	0.06	H→L 99.0%	-	**2.88**	1.50	825	0.05	H→L 98.8%	-
S_2 ([1]LE)	ACN	3.70	3.47	358	0.21	H→L+1 97.8%	-	3.03	2.72	456	0.11	H-1→L 98.0%	-
		**BP-DA**						**BP-TA**					
[1]ICT	Tol	2.56	2.35	527	0.30	H→L 97.0%	500	2.52	2.37	523	0.57	H→L 96.5%	500
[1]ICT	ACN	**2.52**	2.34	529	0.60	H→L 95.8%	548*	**2.44**	2.25	551	0.59	H→L 95.1%	608*
[1]TICT	ACN	**3.01**	1.56	796	0.10	H→L 90.7%<br>H-1→L 8.2%	644	**2.88**	1.75	709	1.01	H→L 93.7%<br>H-1→L 3.9%	-
S_2 ([1]LE)	ACN	**2.98**	2.78	447	0.75	H-1→L 95.9%	-	**2.90**	2.71	458	0.77	H-1→L 95.1%<br>H→L 3.7%	462

Table S9. Computed emission properties of BOPAMs at S_1 and S_2 ([1]LE, [1]HLCT, and [1]ICT) state and [1]TICT state (twisted configuration), which was optimized by TD-DFT method using PBE0/6-31+g(d) level theory in IEFPCM model for acetonitrile. Adiabatic energy difference between S_1 and [1]TICT state (ΔE); vertical energy (E_{vt}); emission wavelength (λ_{ems}) (nm); oscillator strength (f).

	S_1					[1]TICT				$\Delta E_{TICT-S1}$ (eV)
	State	E_{vt} (eV)	λ_{ems} (nm)	f	transition	E_{vt} (eV)	λ_{ems} (nm)	f	transition	
**BP-OM**	[1]ICT	2.29	542	0.51	H→L 98.8%	1.02	1216	0.01	H→L 98.8%	-0.04
**aB-BP**	[1]LE	2.39	519	1.09	H→L 98.9%	1.46	849	0.03	H→L 94.7%<br>H-1→L 3.8%	0.52
**bB-BP**	[1]HLCT	2.51	494	0.68	H→L 99.2%	1.40	883	0.02	H→L 98.8%	0.28
	S_2									ΔE_{S2-S1} (eV)
	State	E_{vt} (eV)	λ_{ems} (nm)	f	transition					
**BP-OM**	[1]LE	2.87	431	0.38	H-1→L 98.2%					0.50
**aB-BP**	[1]HLCT	3.16	392	0.26	H-1→L 98.6%					0.96
**bB-BP**	[1]LE	2.79	445	0.67	H-1→L 98.3%					0.38

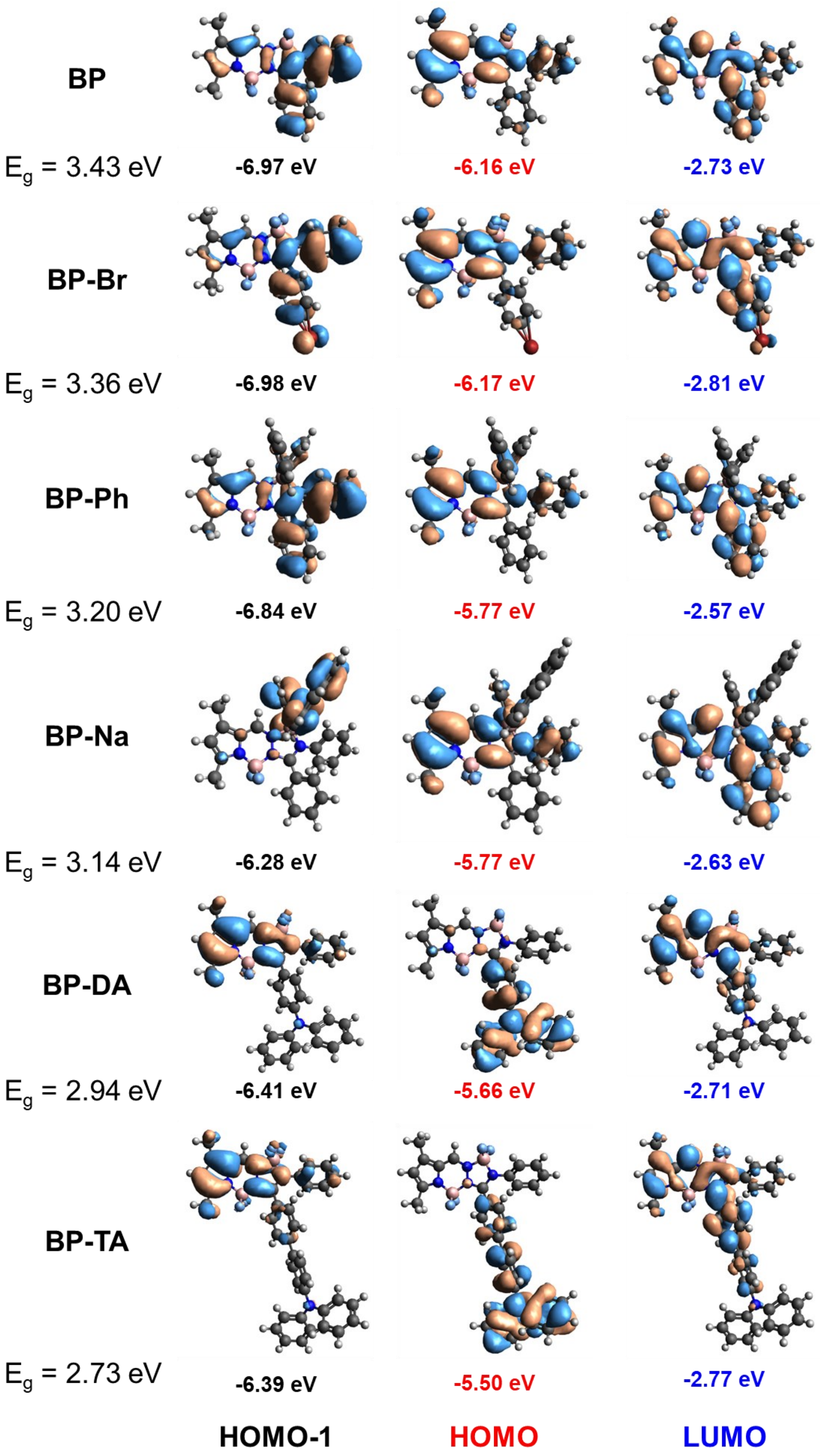

Figure S7. MO images and energy level of BOPAMs at S_1 geometry, which was optimized by TD-DFT using PBE0/6-31+g(d) in IEFPCM for ACN. HOMO-LUMO energy gap (E_g).

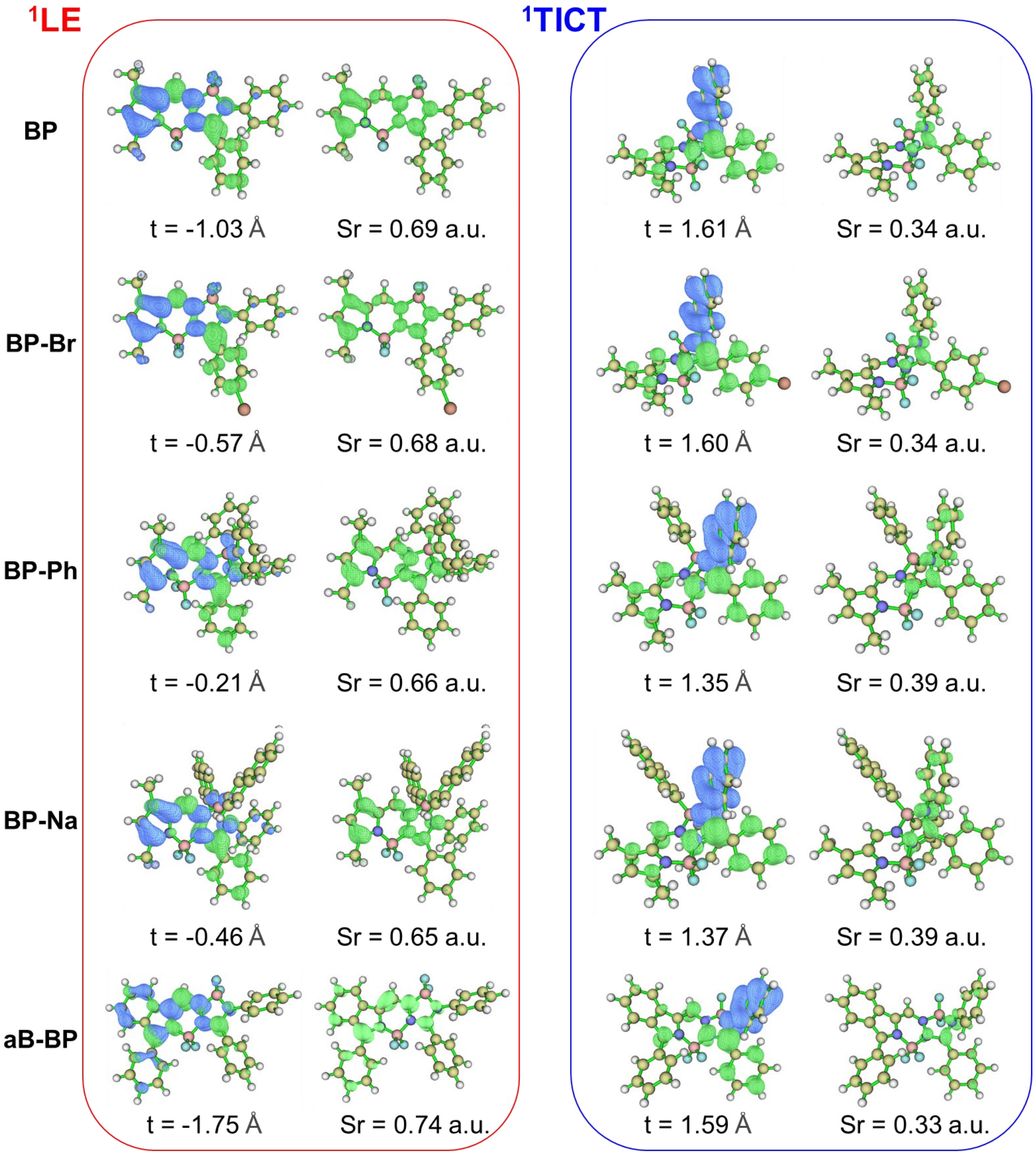

Figure S8. Hole (blue) and electron (green) distribution and its index (t) for ^{1}LE and ^{1}TICT states of **BP**, **BP-Br**, **BP-Ph**, **BP-Na**, and **aB-BP** which was optimized by TD-DFT using PBE0/6-31+g(d) in IEFPCM for ACN. Hole and electron overlap, and its index (Sr) are shown on the right side of hole and electron distribution.

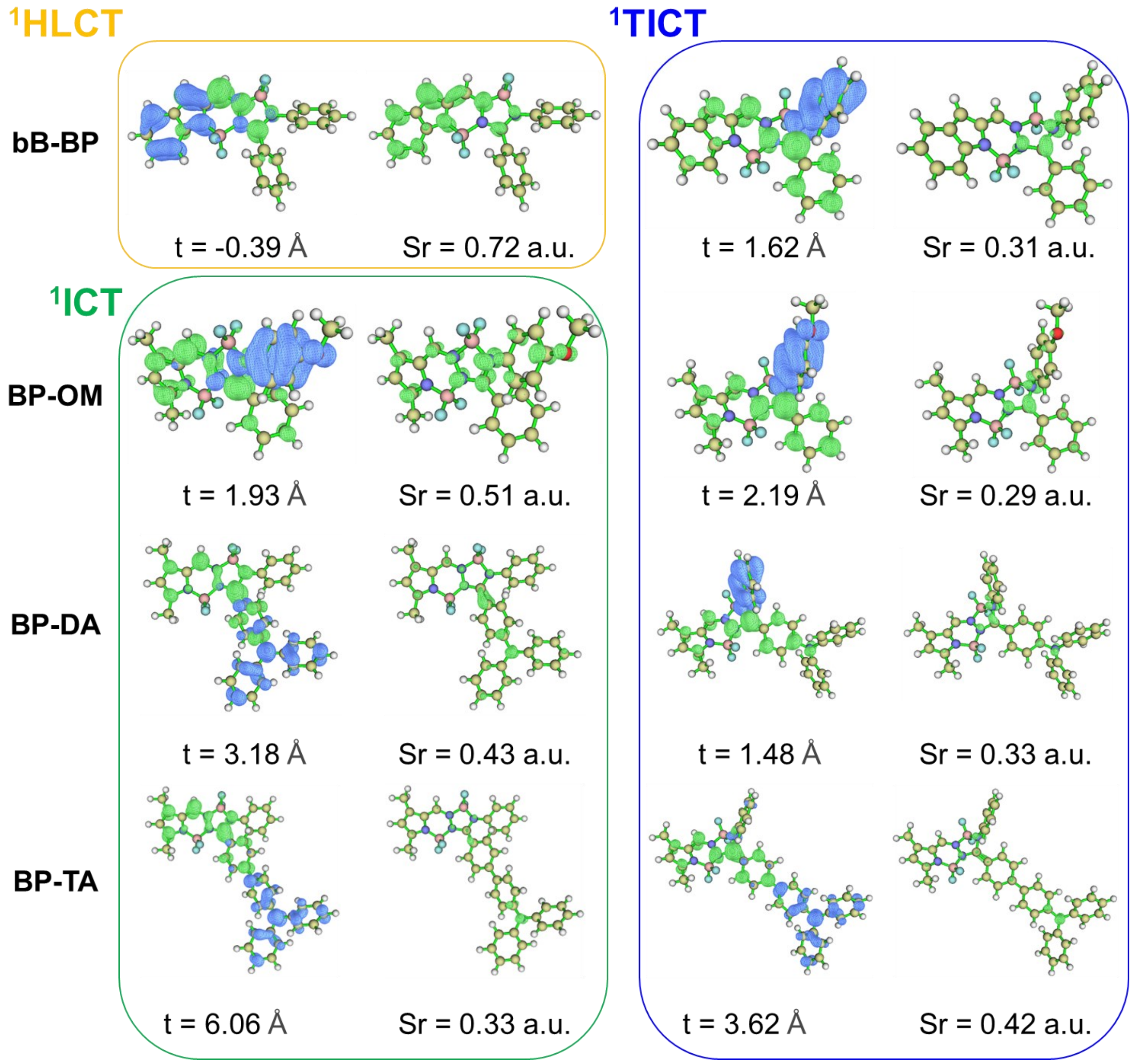

Figure S9. Hole (blue) and electron (green) distribution and its index (t) for ^{1}HLCT, ^{1}ICT and ^{1}TICT states of **bB-BP**, **BP-OM**, **BP-DA**, and **BP-TA** which was optimized by TD-DFT/PBE0/6-31+g(d) in IEFPCM for ACN. Hole and electron overlap, and its index (Sr) are shown on the right side of hole and electron distribution.

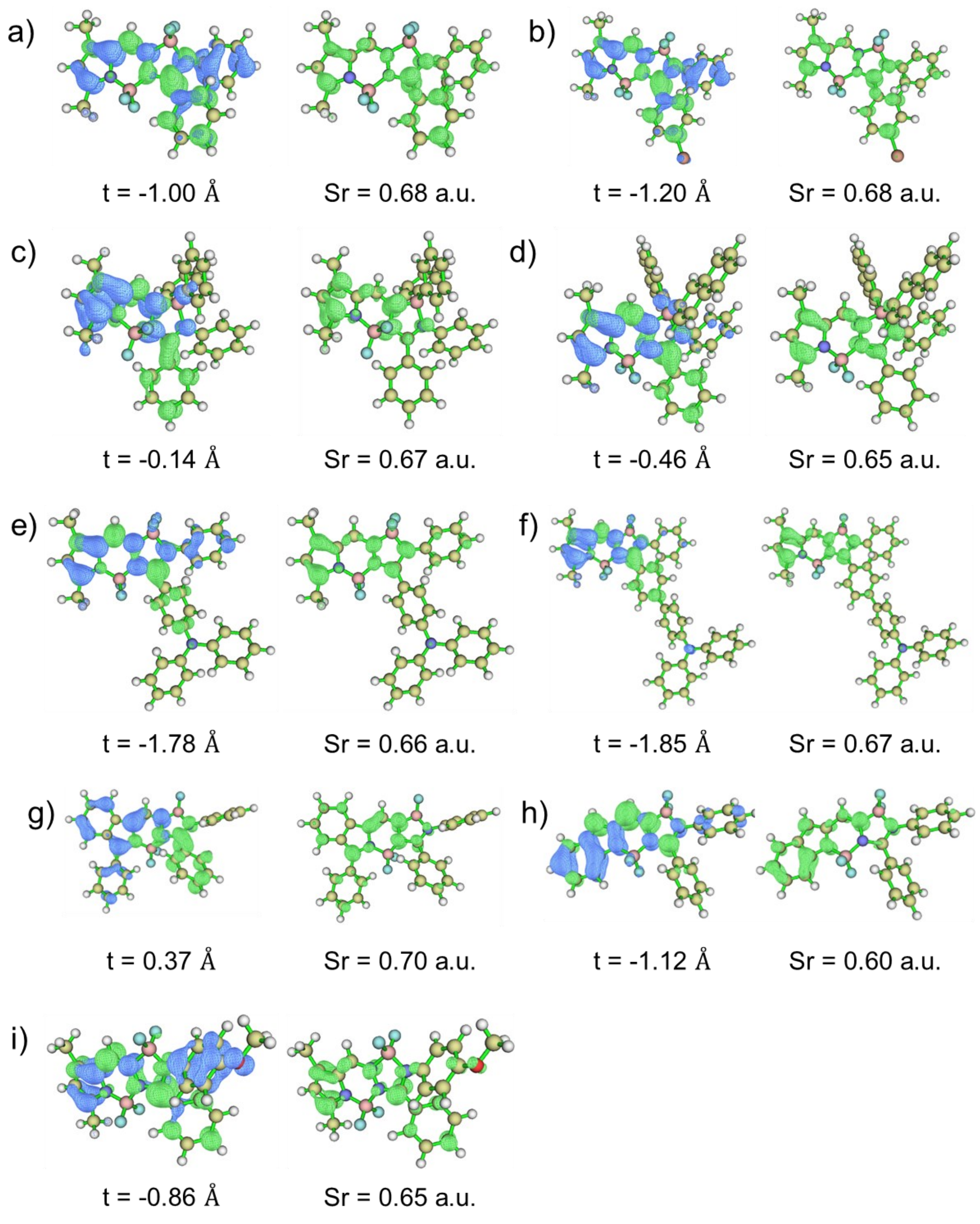

Figure S10. Hole (blue) and electron (green) distribution and its index (t) for S_2 states of a) **BP**, b) **BP-Br**, c) **BP-Ph**, d) **BP-Na**, e) **BP-DA**, f) **BP-TA**, g) **aB-BP**, h) **bB-BP**, and i) **BP-OM** which was optimized by TD-DFT/PBE0/6-31+g(d) in IEFPCM for ACN. Hole and electron overlap, and its index (Sr) are shown on the right side of hole and electron distribution.

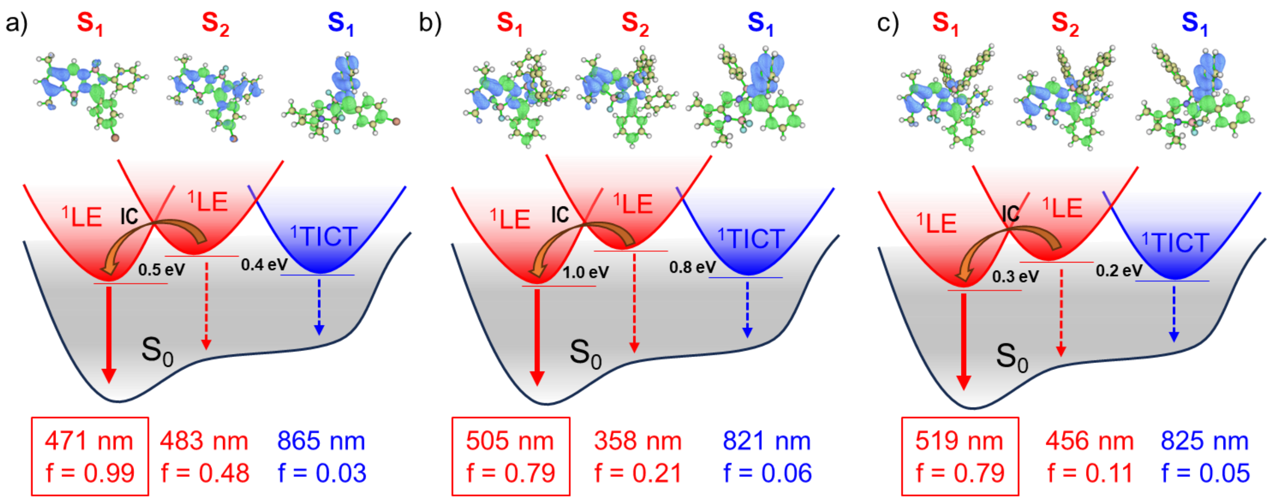

Figure S11. Proposed mechanism for $S_1 \rightarrow {}^1$TICT transition of a) **BP-Br**, b) **BP-Ph**, and c) **BP-Na** illustrated by hole (blue)-electron (green) distribution and oscillator strength (f).

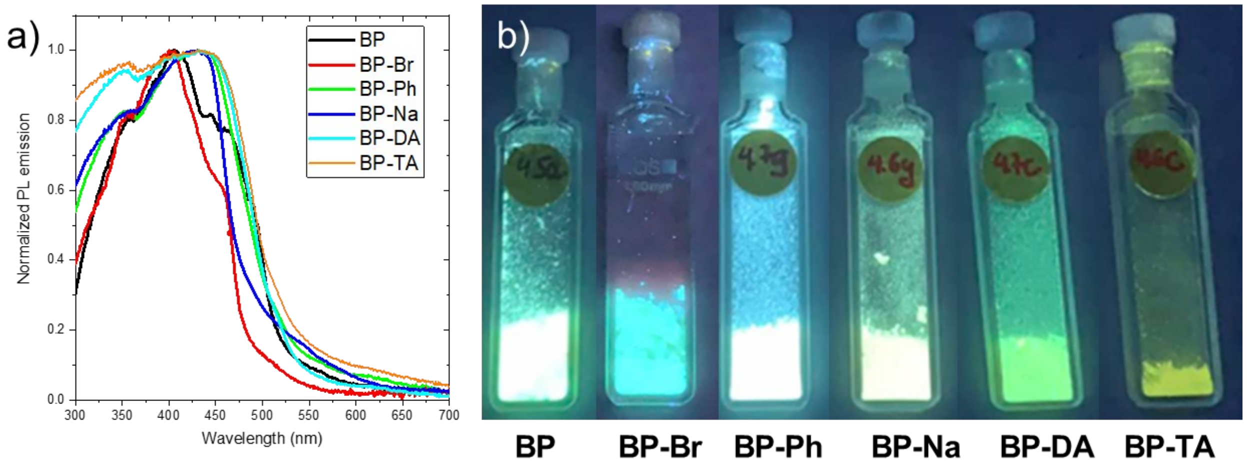

Figure S12. a) Normalized UV-Vis absorption spectra and b) Fluorescence image (excitation at 365 nm) of **BP**, **BP-Br**, **BP-Ph**, **BP-Na**, **BP-DA** and **BP-TA** in solid state.

5. Structural characterization

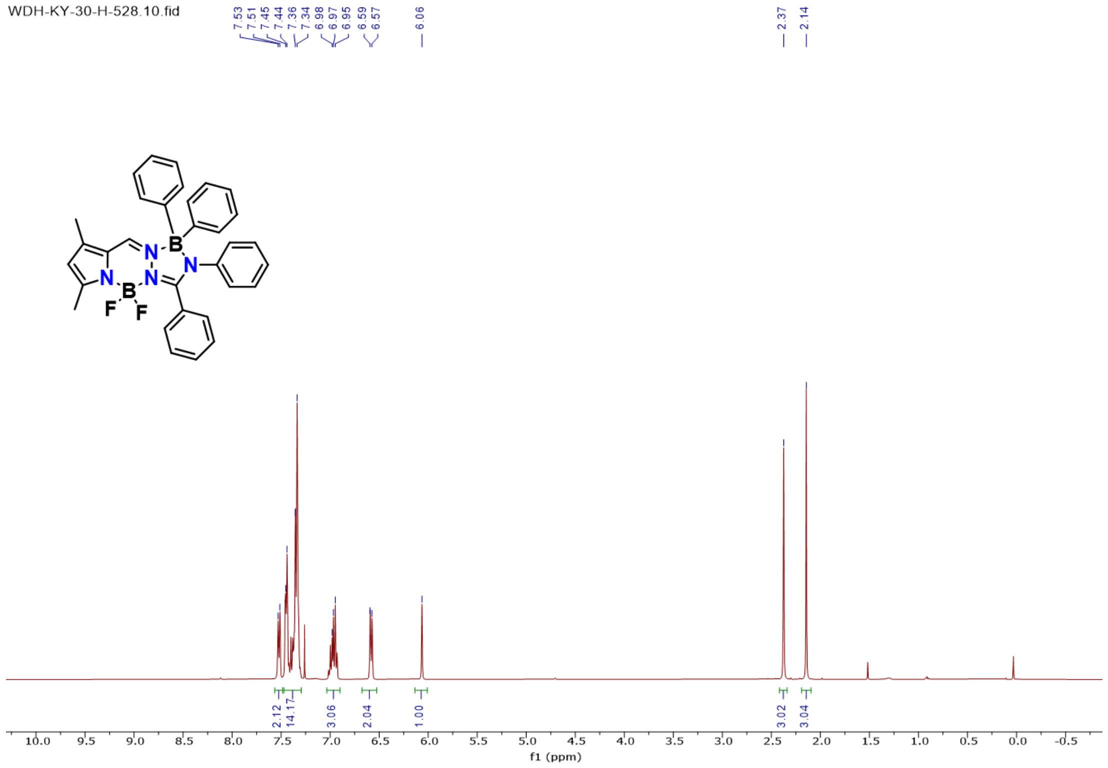

Figure S13. ^{1}H NMR of **BP-Ph**

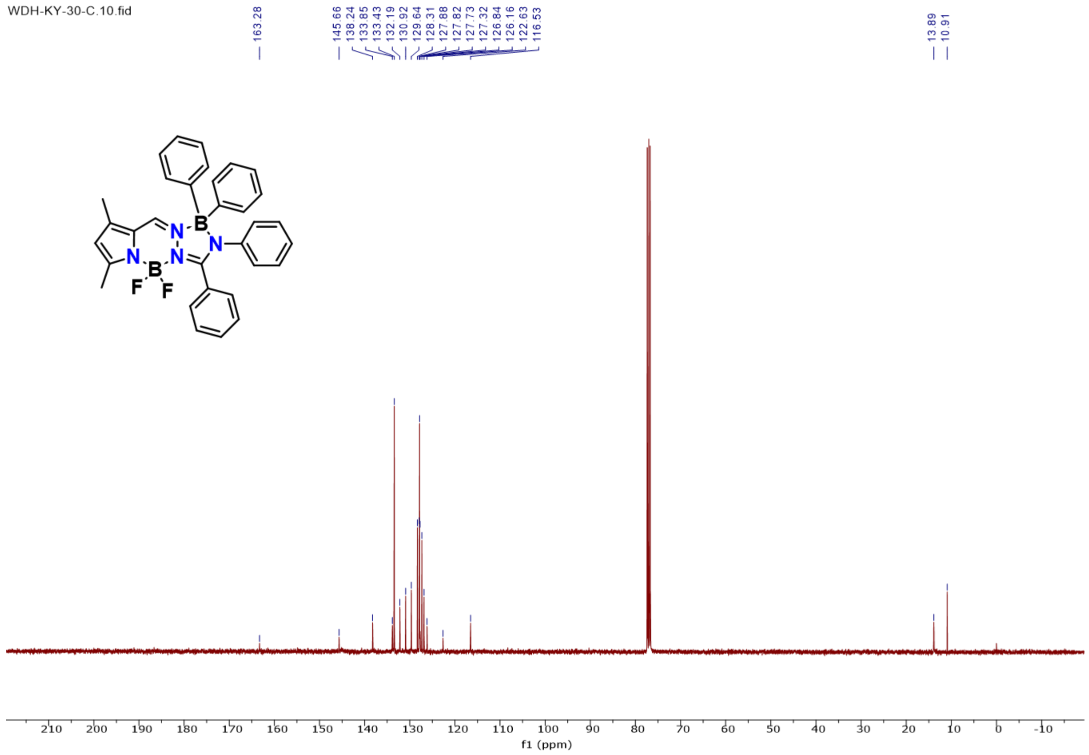

Figure S14. ^{13}C NMR of **BP-Ph**

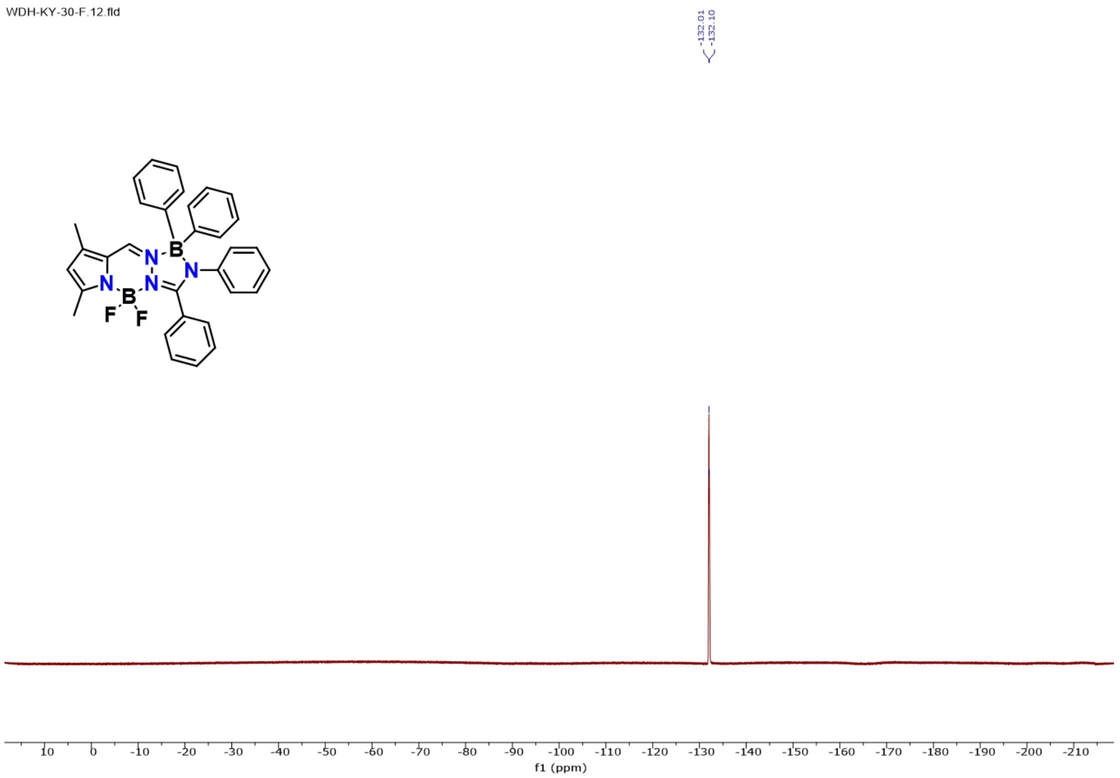

Figure S15. ^{19}F NMR of **BP-Ph**

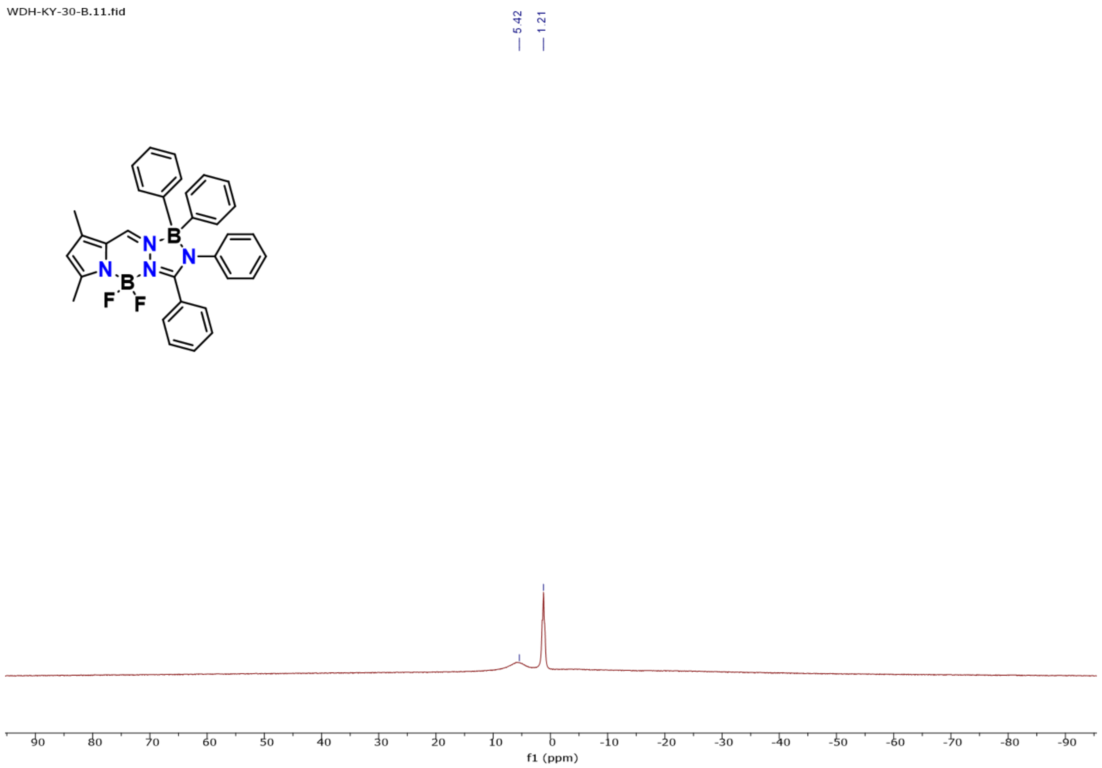

Figure S16. ^{11}B NMR of **BP-Ph**

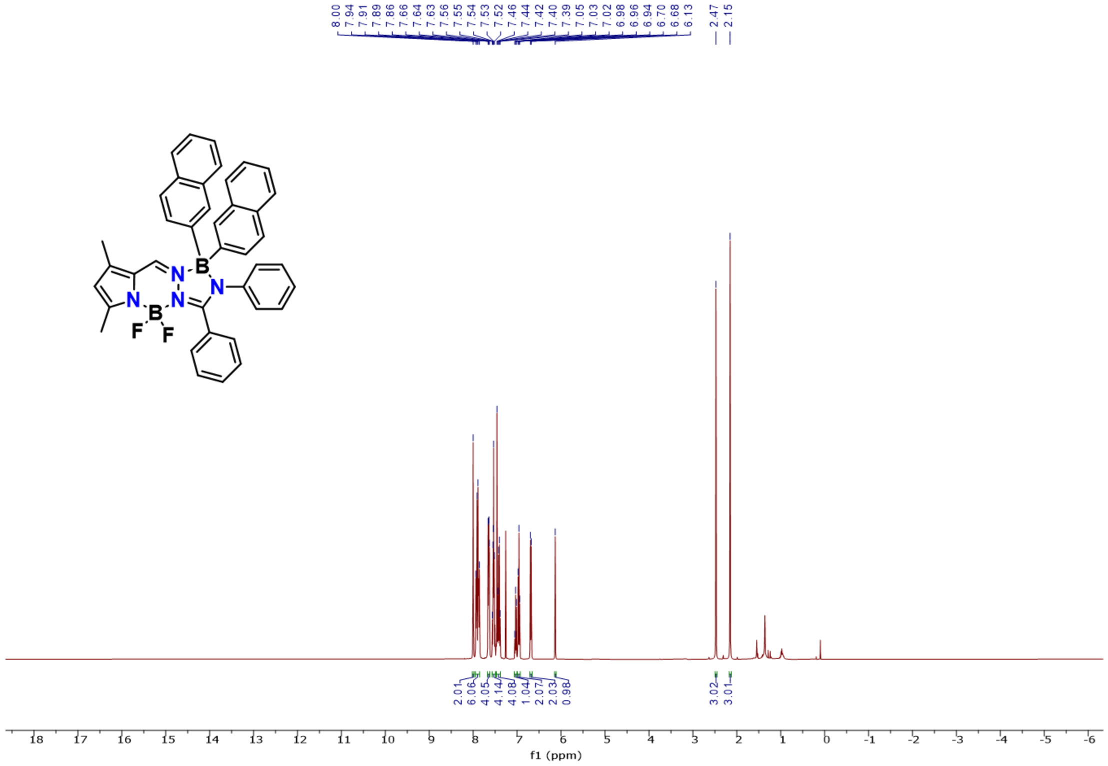

Figure S17. ^{1}H NMR of **BP-Na**

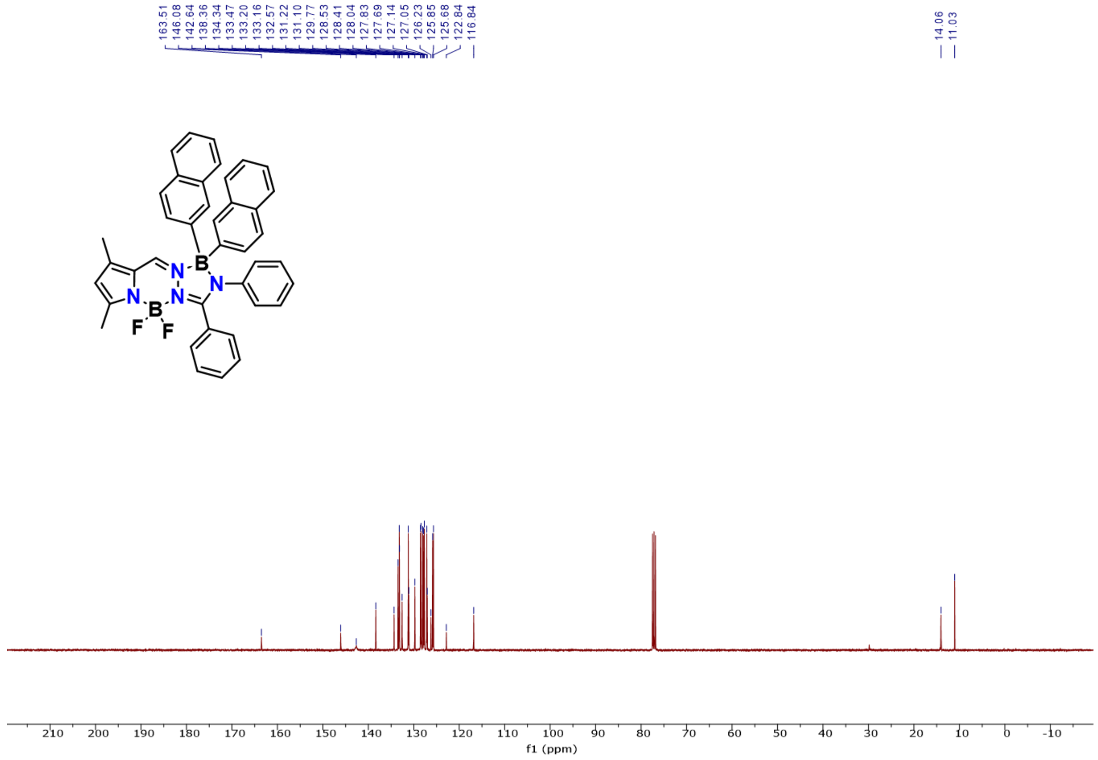

Figure S18. ^{13}C NMR of **BP-Na**

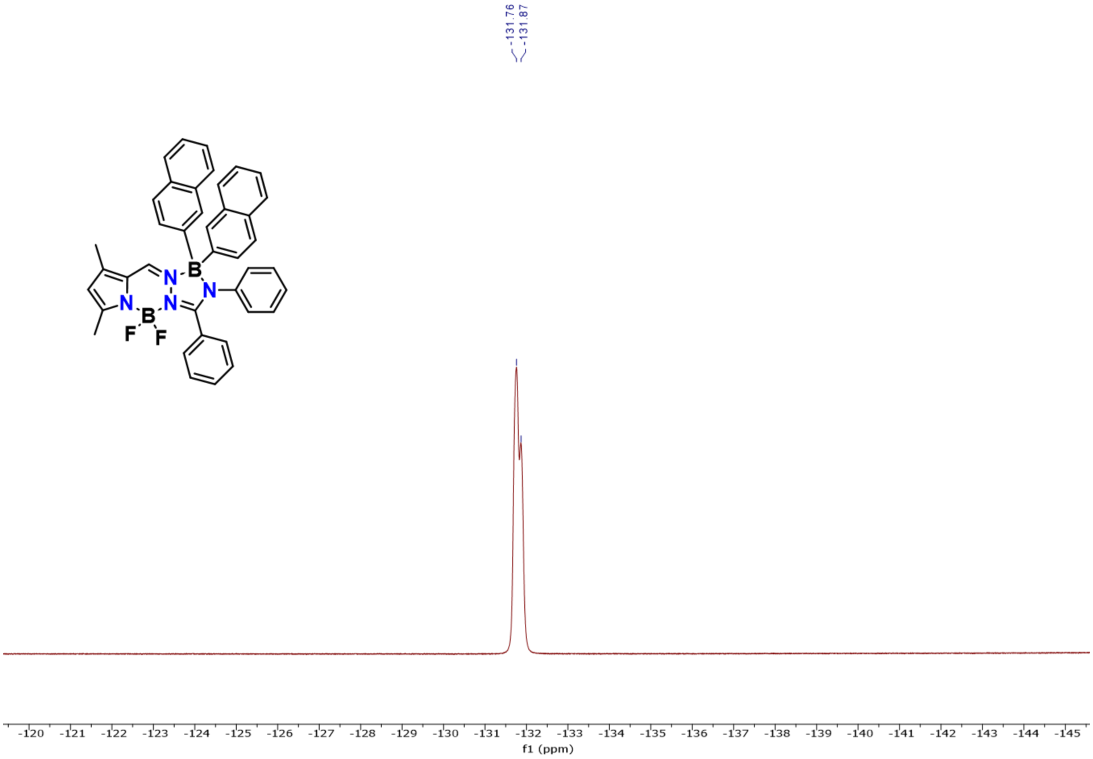

Figure S19. ^{19}F NMR of **BP-Na**

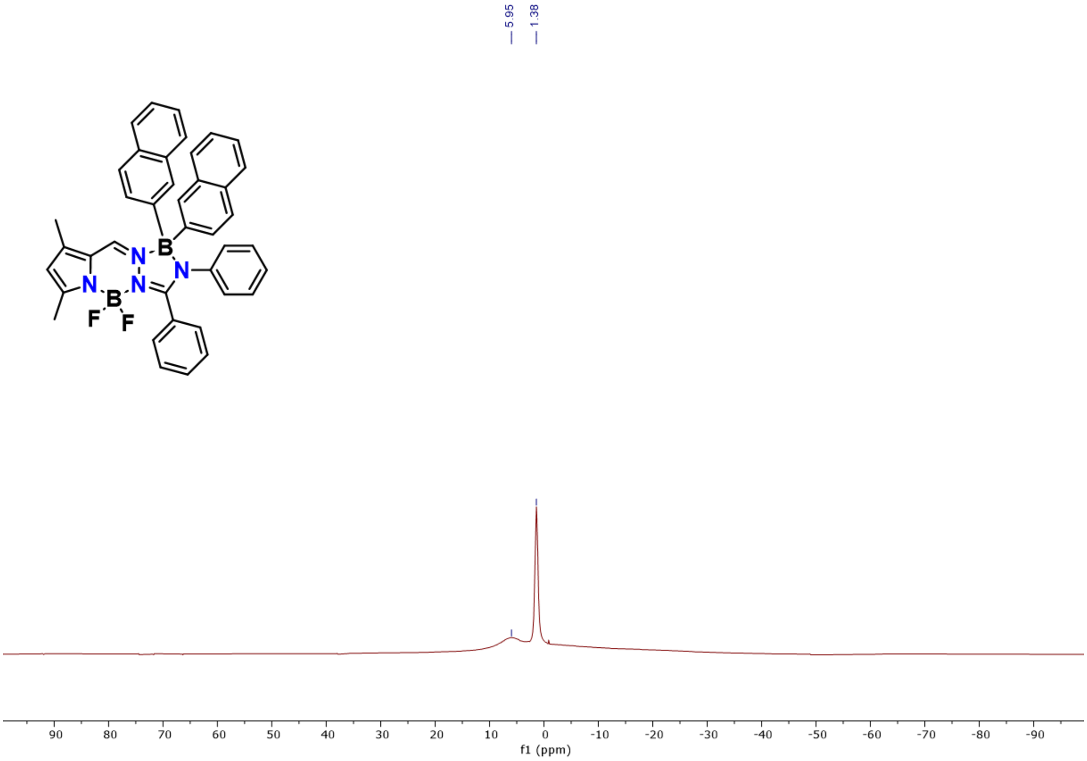

Figure S20. ^{11}B NMR of **BP-Na**

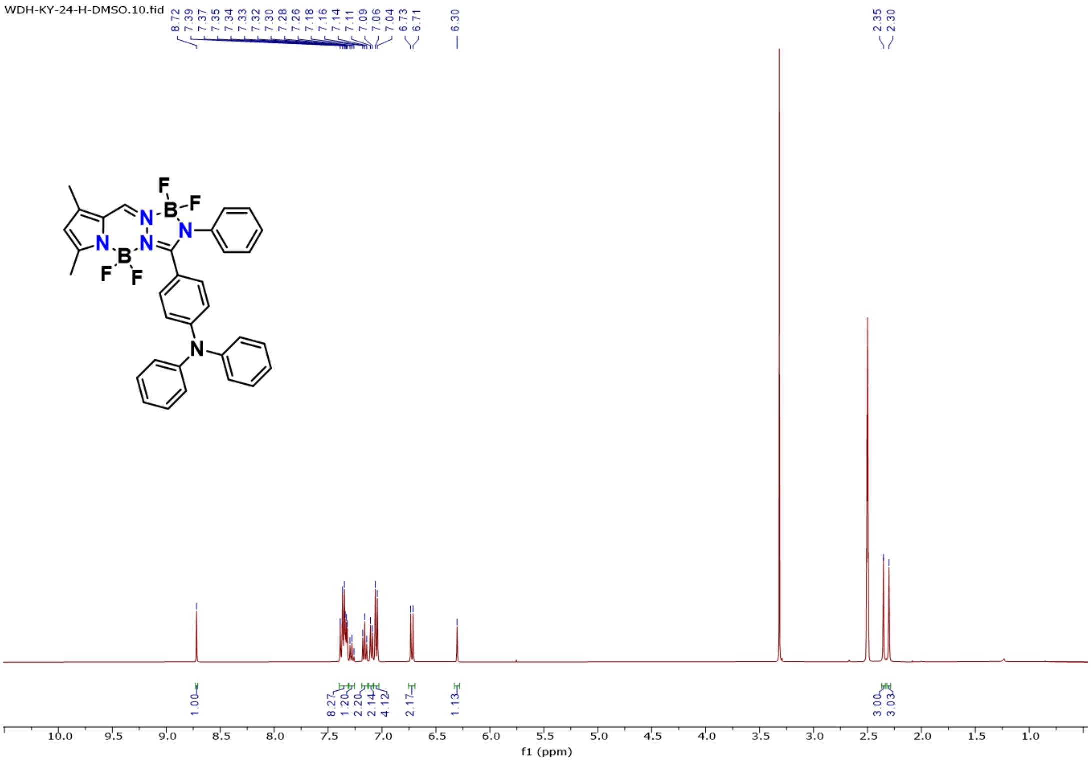

Figure S21. ^{1}H NMR of **BP-DA**

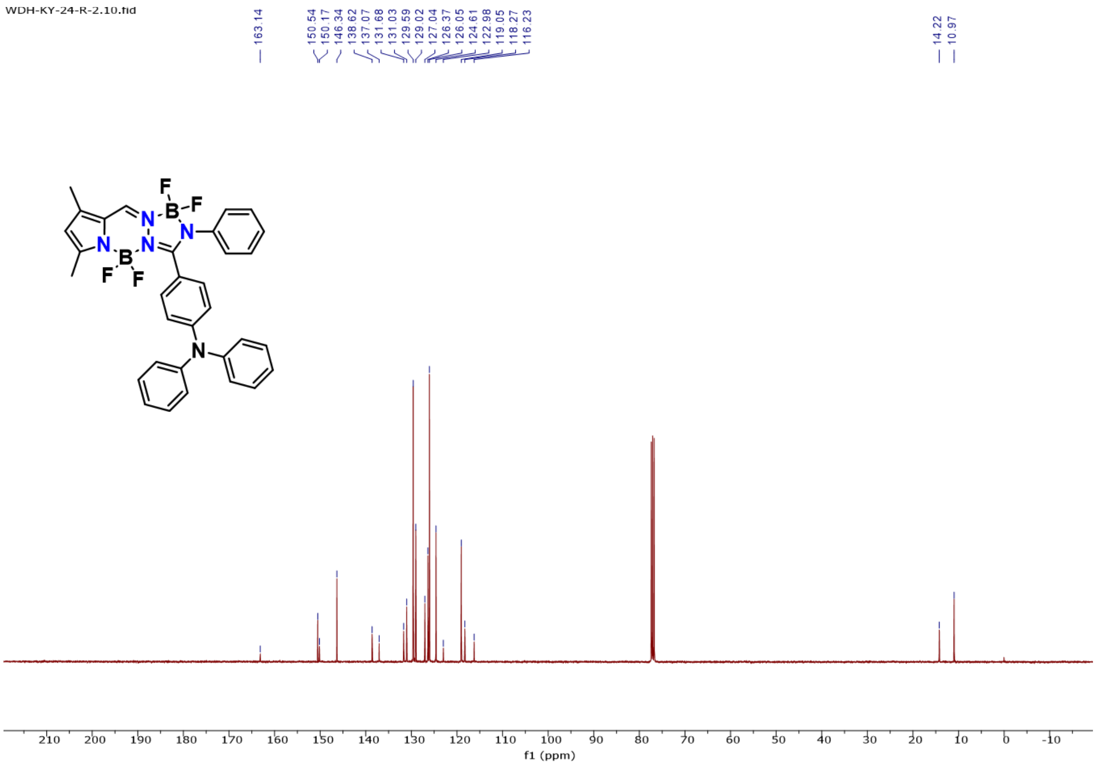

Figure S22. ^{13}C NMR of **BP-DA**

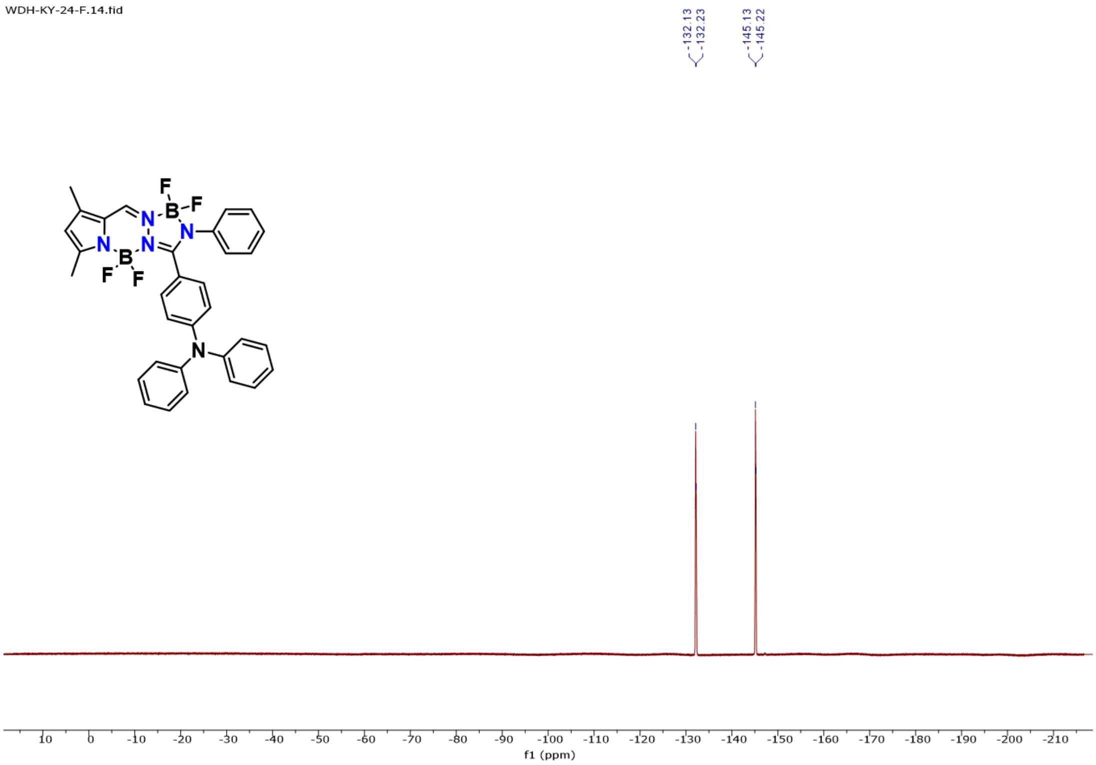

Figure S23. ^{19}F NMR of **BP-DA**

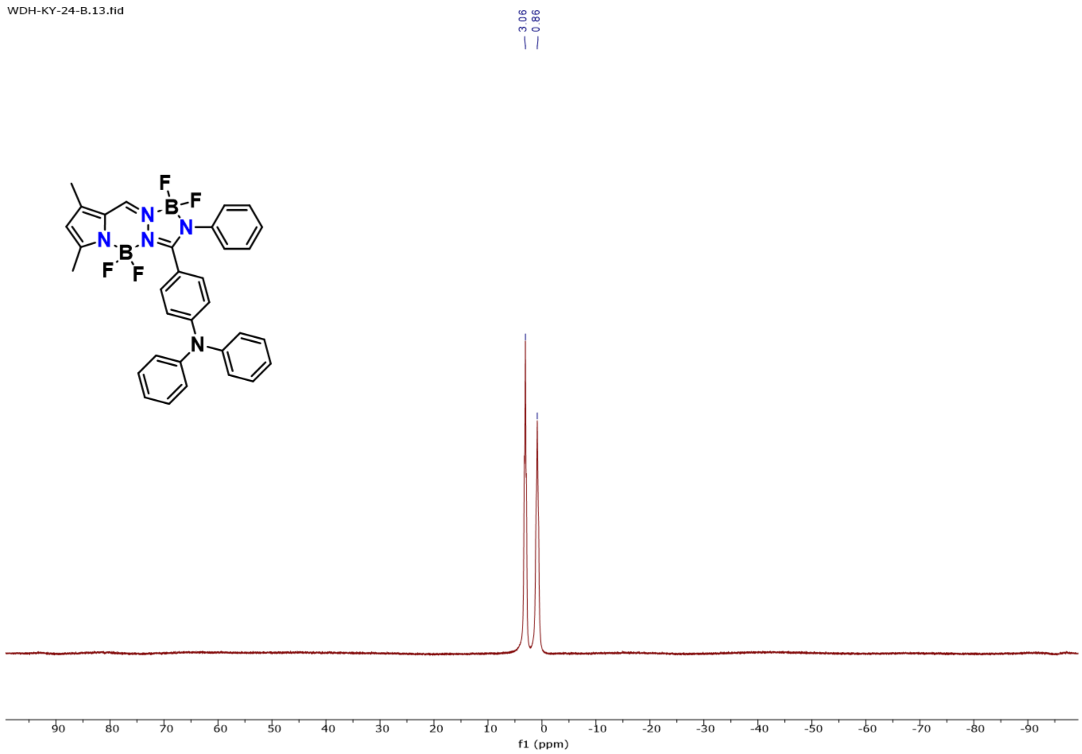

Figure S24. ^{11}B NMR of **BP-DA**

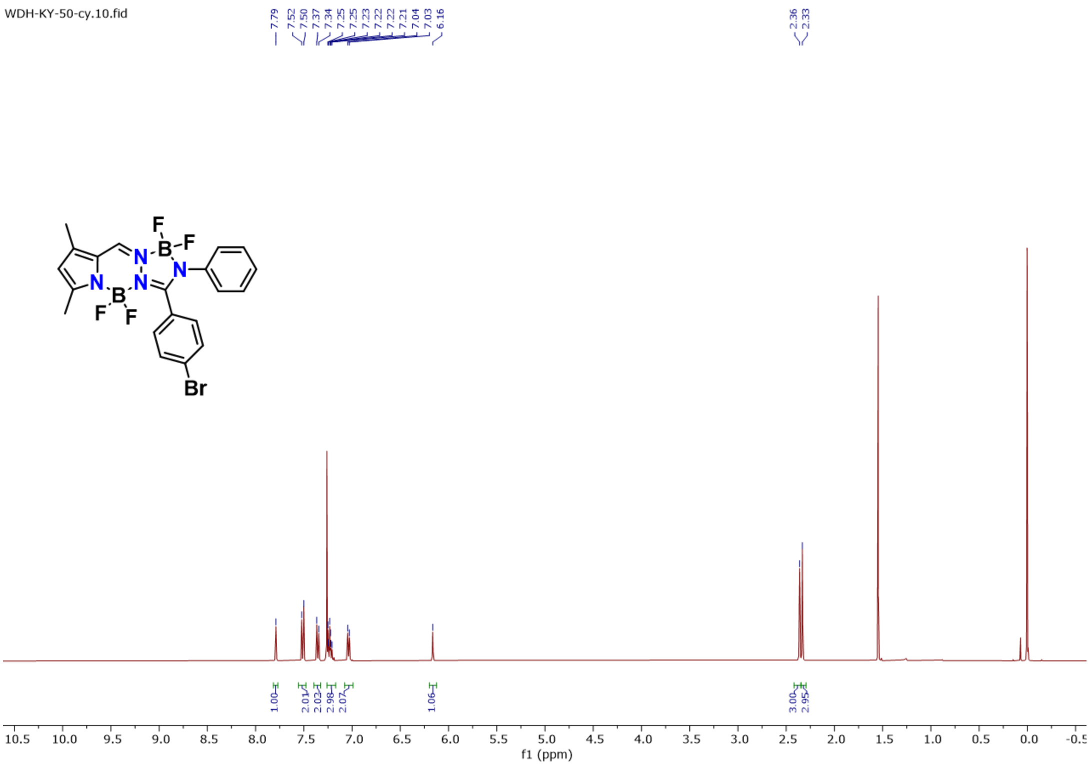

Figure S25. ^{1}H NMR of **BP-Br**

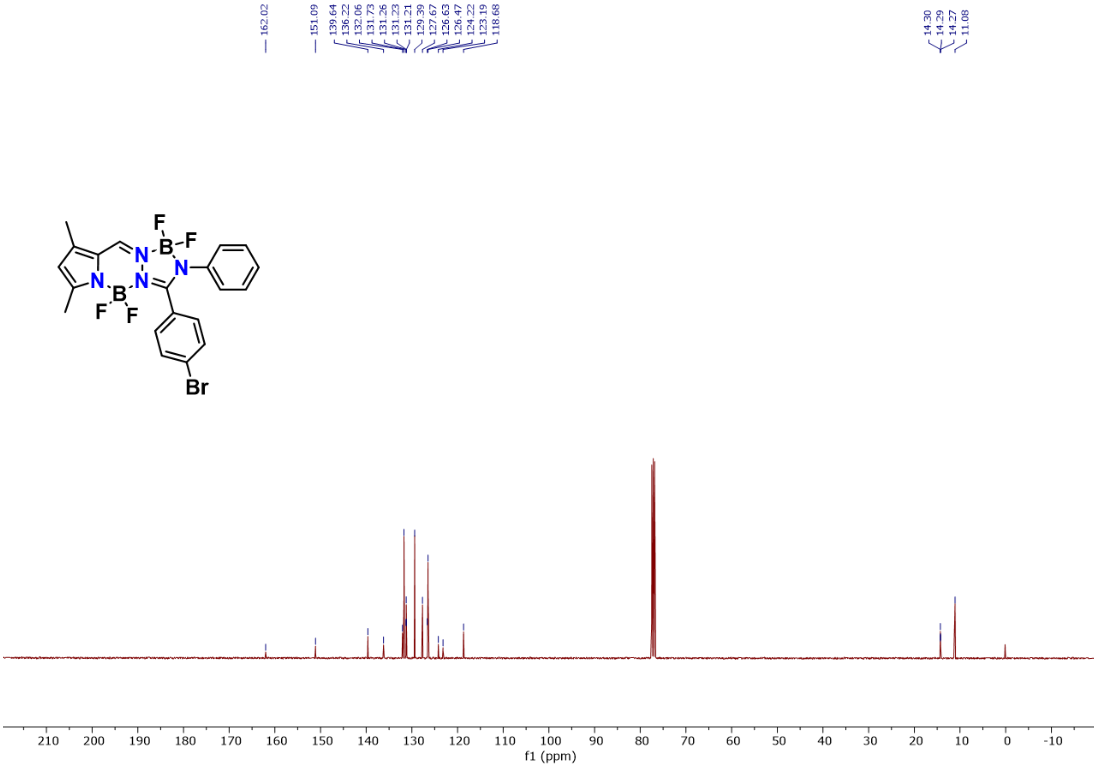

Figure S26. ^{13}C NMR of **BP-Br**

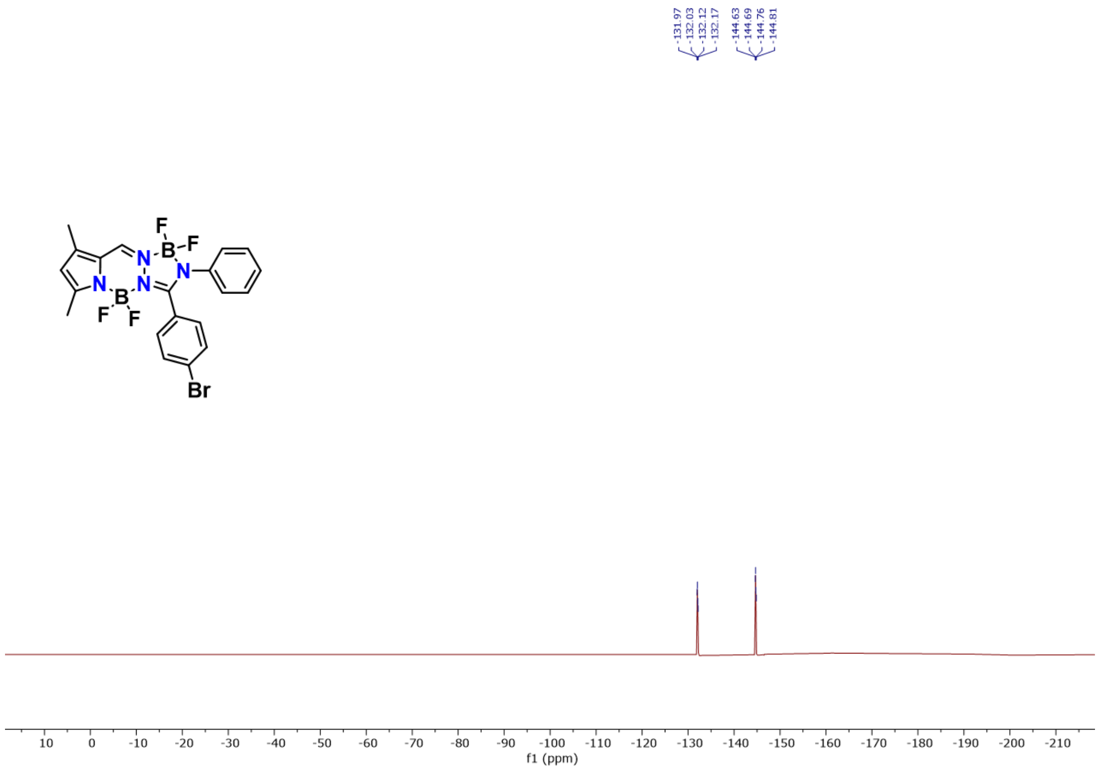

Figure S27. ^{19}F NMR of **BP-Br**

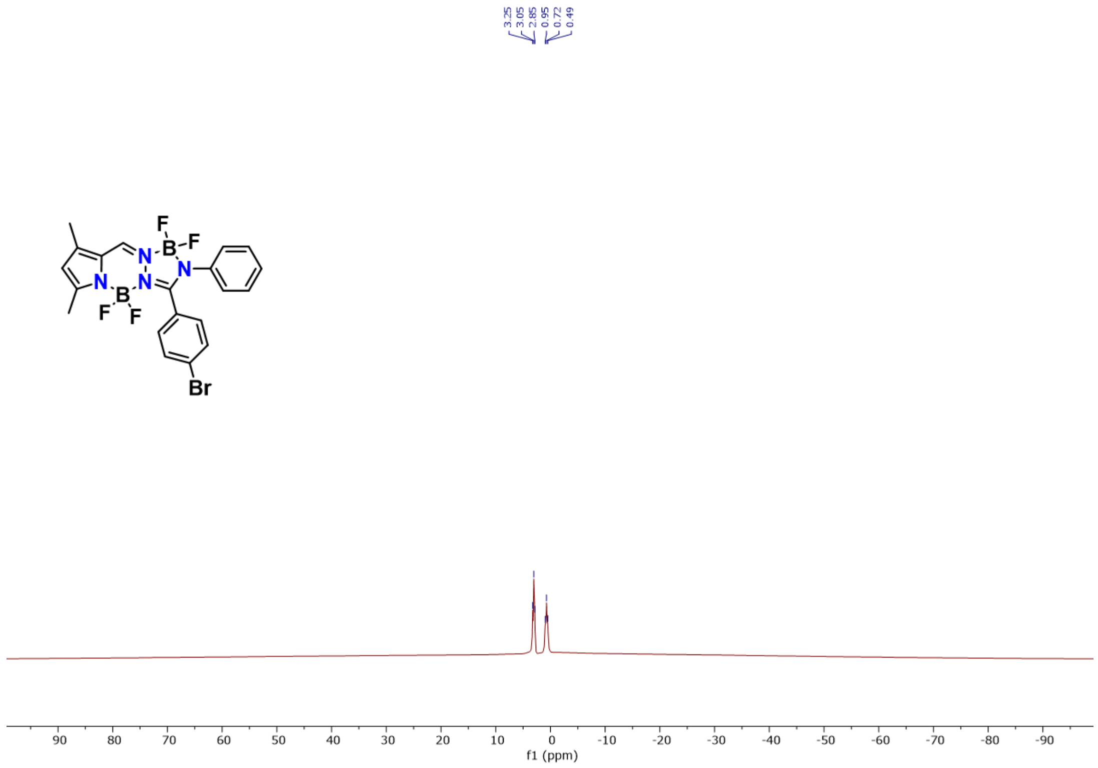

Figure S28. ^{11}B NMR of **BP-Br**

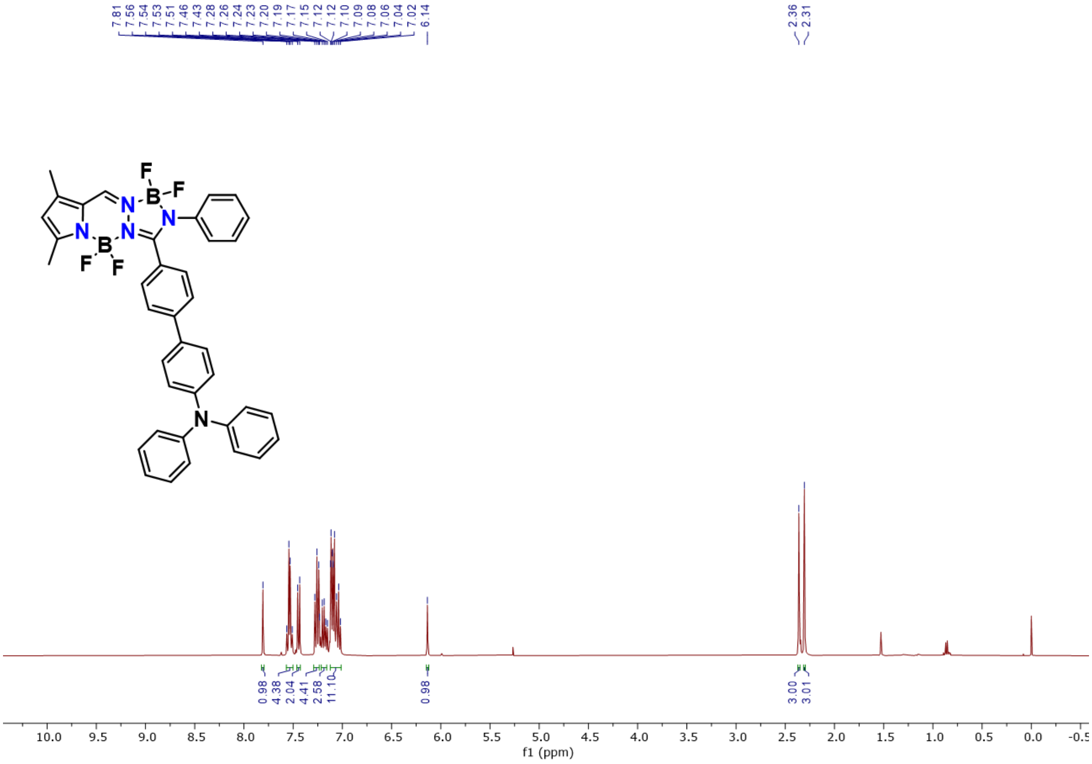

Figure S29. ^{1}H NMR of **BP-TA**

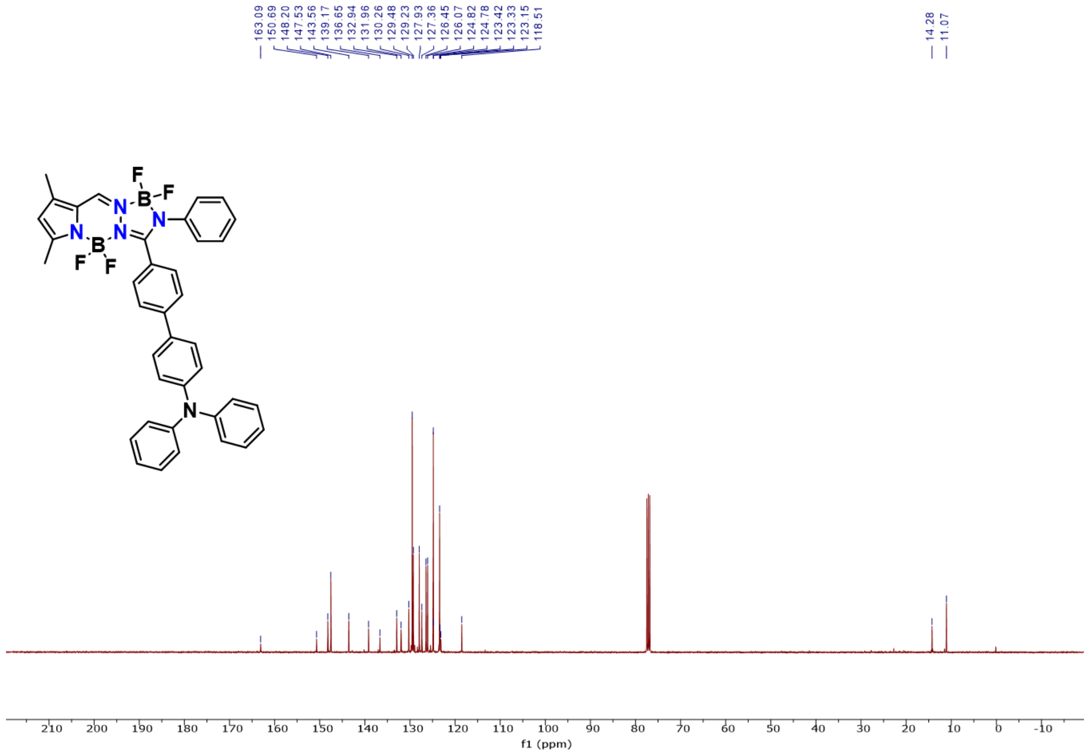

Figure S30. ^{13}C NMR of **BP-TA**

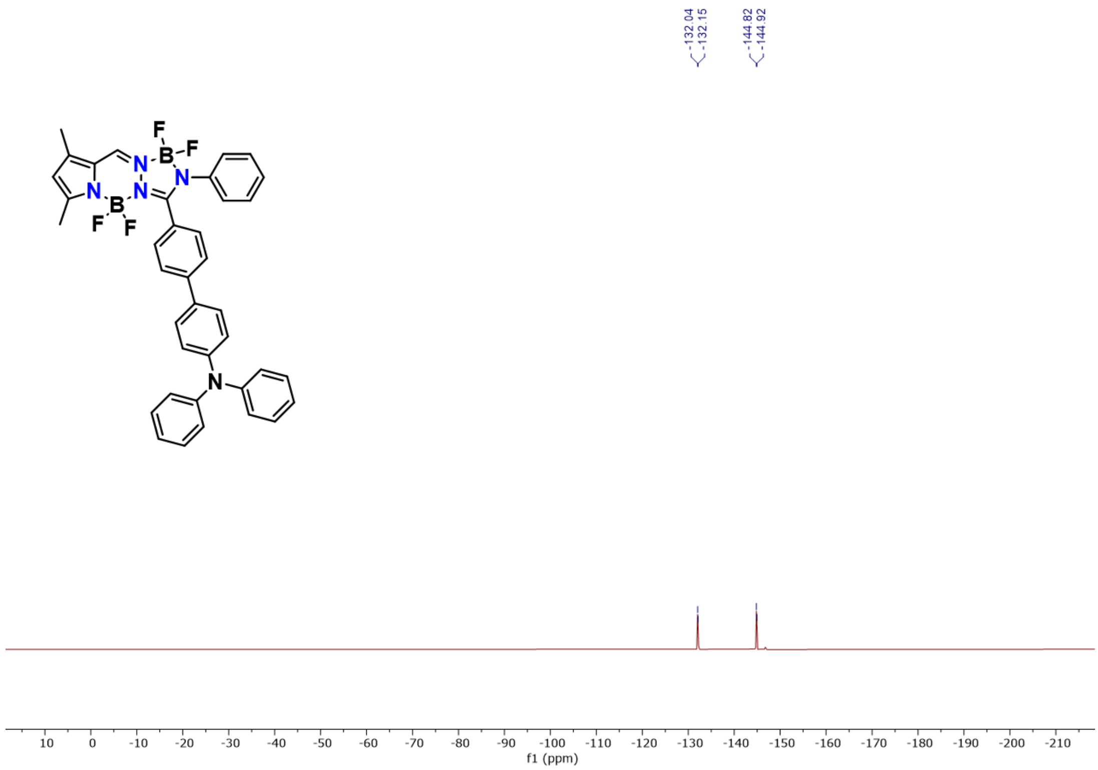

Figure S31. ^{19}F NMR of **BP-TA**

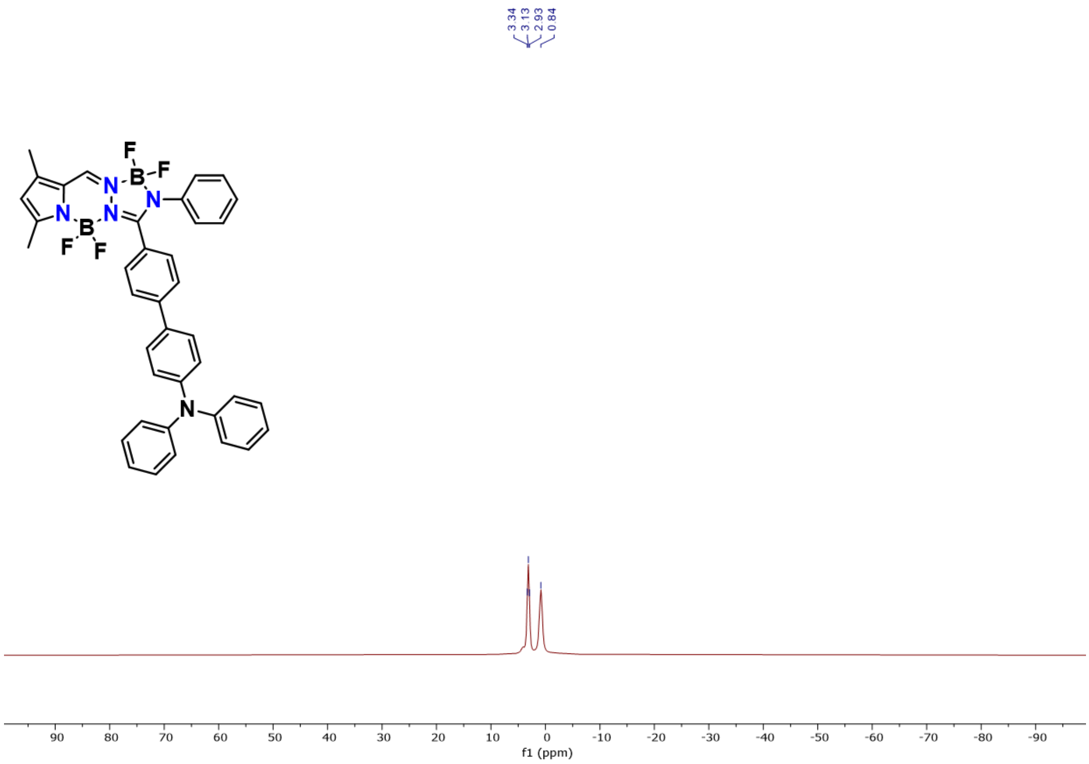

Figure S32. ^{11}B NMR of **BP-TA**

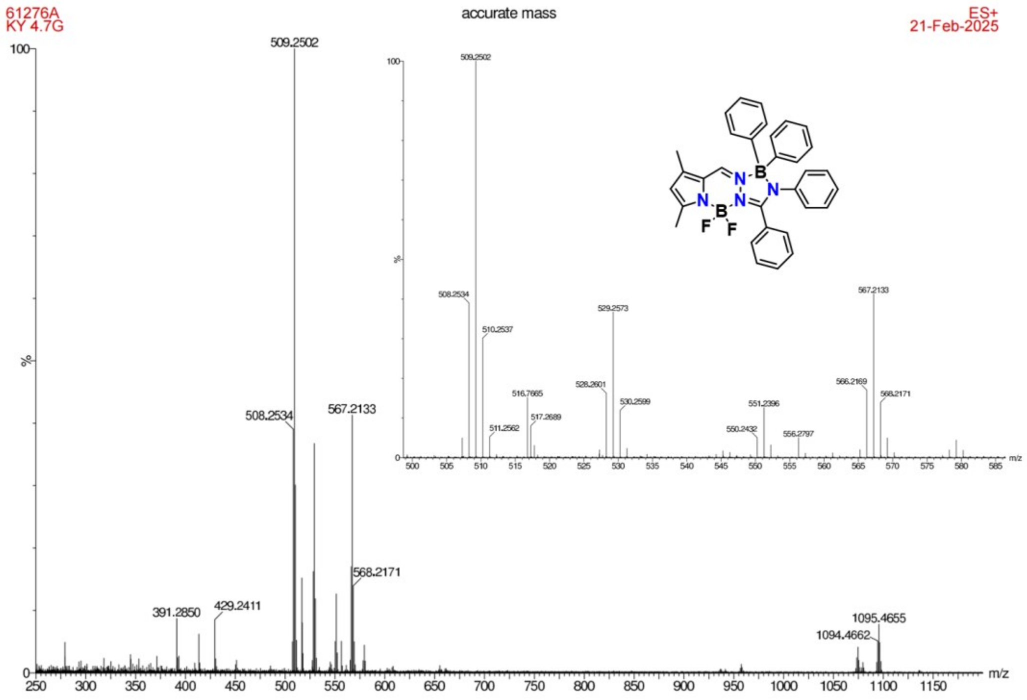

Figure S33. HRMS spectrum of **BP-Ph**

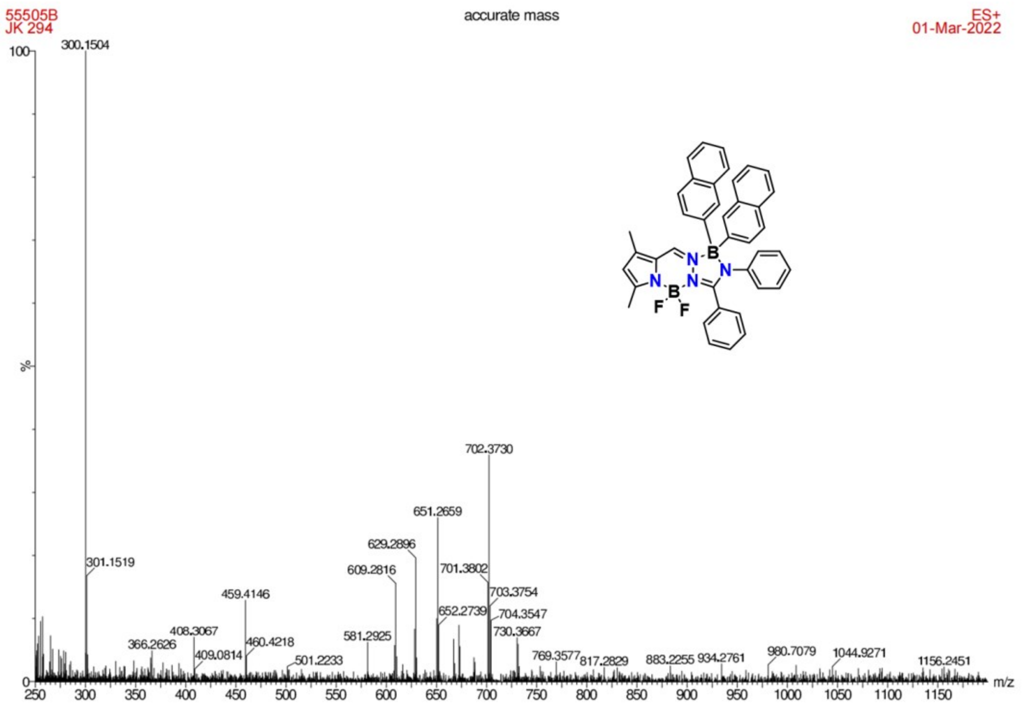

Figure S34. HRMS spectrum of **BP-Na**

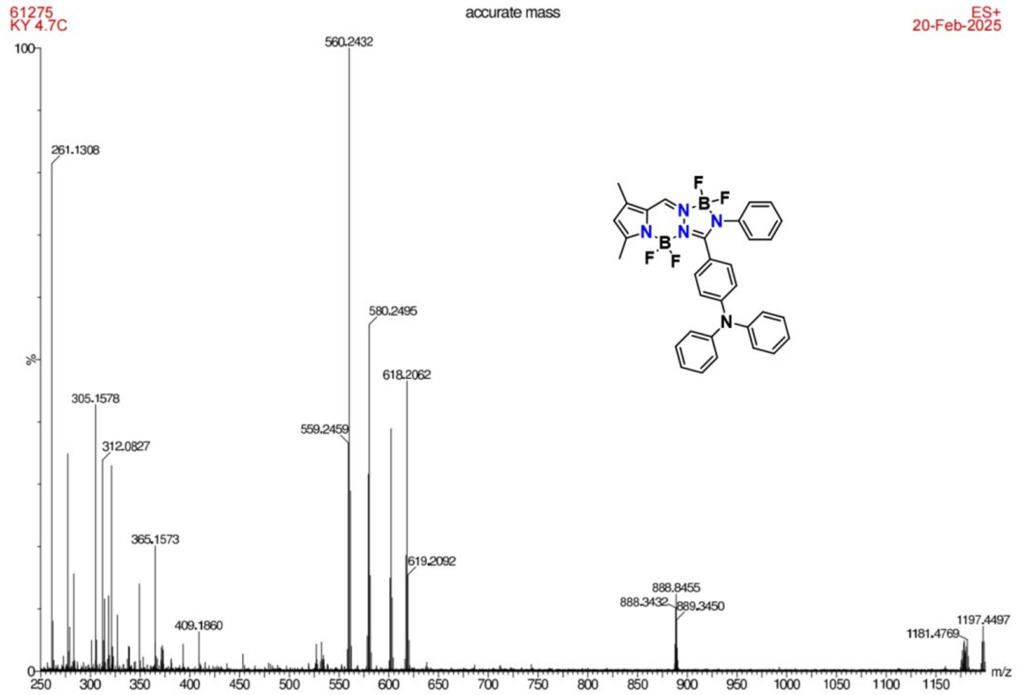

Figure S35. HRMS spectrum of **BP-DA**

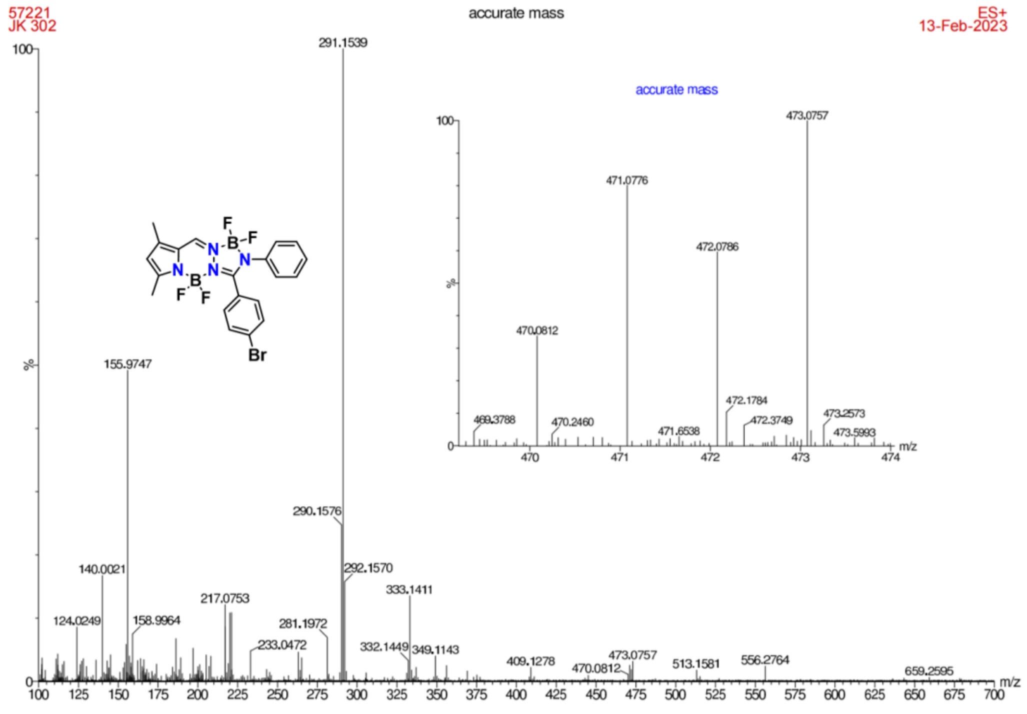

Figure S36. HRMS spectrum of **BP-Br**

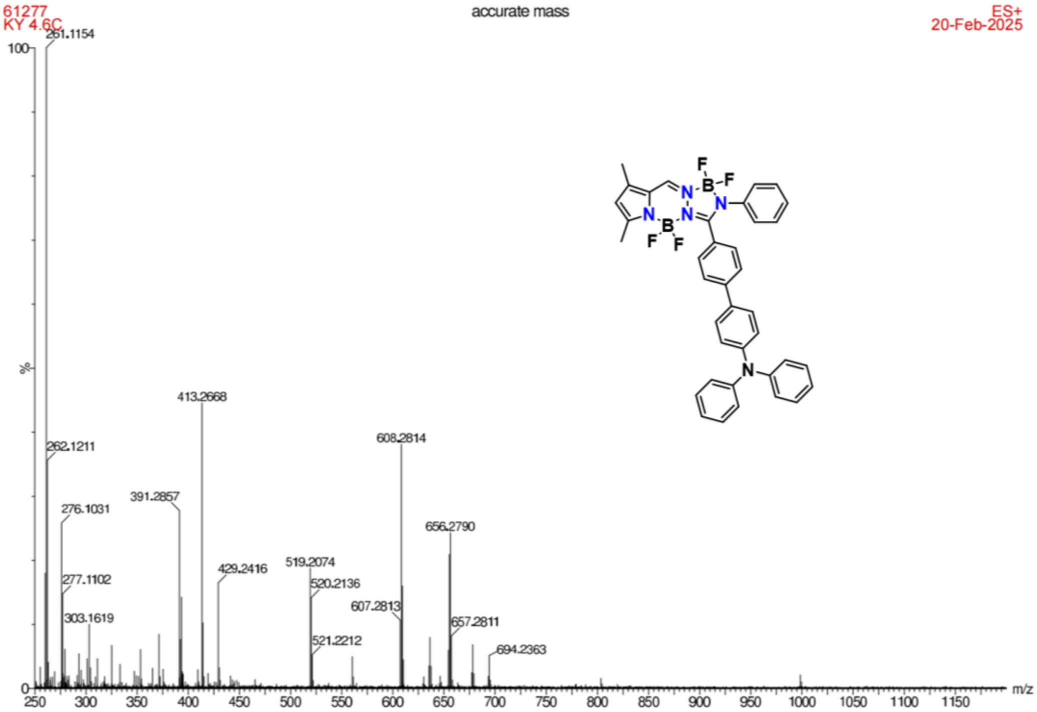

Figure S37. HRMS spectrum of **BOPAM-TA**

Table S10. Crystal data and structure refinement for **BP-Br, BP-Ph, BP-Na, BP-DA** and **BP-TA.**

Compound	**BP-Br**	**BP-Ph**	**BP-Na**	**BP-DA**	**BP-TA**
Empirical formula	$C_{20}H_{17}B_2BrF_4N_4$	$C_{32}H_{28}B_2F_2N_4$	$C_{40}H_{32}B_2F_2N_4$	$C_{32}H_{27}B_2F_4N_5$	$C_{38}H_{31}B_2F_4N_5$
Formula weight	490.90	528.20	628.32	579.20	655.30
Temperature/K	294(2)	294(2)	294(2)	294(2)	294(2)
Crystal system	orthorhombic	monoclinic	triclinic	triclinic	triclinic
Space group	*Pbca*	$P2_1/n$	*P*-1	*P*-1	*P*-1
a/Å	18.2042(11)	12.8019(6)	12.3908(4)	8.8168(3)	8.3774(3)
b/Å	10.9107(5)	10.0014(4)	14.9059(4)	11.3696(4)	13.2992(5)
c/Å	20.9493(7)	22.4384(10)	19.5103(6)	14.4819(5)	15.7592(5)
α/°	90	90	84.308(2)	85.122(3)	85.861(3)
β/°	90	96.385(4)	87.599(3)	79.900(3)	78.384(3)
γ/°	90	90	70.661(3)	87.216(3)	78.253(3)
Volume/Å^3	4160.9(3)	2855.1(2)	3383.22(18)	1423.20(9)	1682.90(10)
Z	8	4	4	2	2
ρ_{calc} g/cm^3	1.567	1.229	1.234	1.352	1.293
μ/mm^{-1}	2.026	0.081	0.080	0.099	0.092
F(000)	1968.0	1104.0	1312.0	600.0	680.0
Crystal size/mm^3	0.45 × 0.25 × 0.2	0.5 × 0.3 × 0.25	0.35 × 0.25 × 0.2	0.4 × 0.4 × 0.2	0.25 × 0.2 × 0.2
Radiation (Å)	Mo Kα (λ = 0.71073)	Mo Kα (λ = 0.71073)	Mo Kα (λ = 0.71073)	Mo Kα (λ = 0.71073)	Mo Kα (λ = 0.71073)
2Θ range for data collection/°	7.008 to 52.73	6.542 to 52.744	6.554 to 52.744	6.528 to 52.744	6.446 to 52.744
Index ranges	-17 ≤ h ≤ 22, -13 ≤ k ≤ 8, -16 ≤ l ≤ 26	-16 ≤ h ≤ 16, -12 ≤ k ≤ 12, -28 ≤ l ≤ 28	-15 ≤ h ≤ 15, -18 ≤ k ≤ 18, -24 ≤ l ≤ 24	-11 ≤ h ≤ 11, -14 ≤ k ≤ 14, -18 ≤ l ≤ 18	-10 ≤ h ≤ 10, -16 ≤ k ≤ 16, -19 ≤ l ≤ 19
Reflections collected	22142	29886	68965	23403	33930
Independent reflections	4252 [R_{int} = 0.0571, R_{sigma} = 0.0502]	5829 [R_{int} = 0.0401, R_{sigma} = 0.0400]	13832 [R_{int} = 0.0530, R_{sigma} = 0.0610]	5797 [R_{int} = 0.0264, R_{sigma} = 0.0239]	6883 [R_{int} = 0.0411, R_{sigma} = 0.0466]
Data/restraints/parameters	4252/0/282	5829/12/364	13832/0/870	5797/0/391	6883/0/444
Goodness-of-fit on F^2	1.035	1.024	1.032	1.045	1.068
Final R indexes [I>=2σ (I)]	R_1 = 0.0625, wR_2 = 0.1347	R_1 = 0.0585, wR_2 = 0.1386	R_1 = 0.0621, wR_2 = 0.1415	R_1 = 0.0415, wR_2 = 0.0945	R_1 = 0.0701, wR_2 = 0.1911
Final R indexes [all data]	R_1 = 0.1129, wR_2 = 0.1579	R_1 = 0.1000, wR_2 = 0.1651	R_1 = 0.1266, wR_2 = 0.1753	R_1 = 0.0563, wR_2 = 0.1037	R_1 = 0.1126, wR_2 = 0.2198
Largest diff. peak/hole / e Å^{-3}	1.21/-1.18	0.25/-0.22	0.23/-0.23	0.21/-0.17	0.48/-0.28
CCDC deposition code	2428797	2428800	2428798	2428801	2428799
